# Response of Early Life‐Stages of Forest‐Forming Seaweeds From Warm‐Edge and Central Populations to Marine Heatwaves

**DOI:** 10.1002/ece3.72998

**Published:** 2026-01-29

**Authors:** Catalina A. Musrri, Georgina Wood, Adriana Vergés, Damon Britton, Catriona L. Hurd, Ezequiel M. Marzinelli

**Affiliations:** ^1^ School of Life and Environmental Sciences The University of Sydney Sydney New South Wales Australia; ^2^ College of Science and Engineering Flinders University Adelaide South Australia Australia; ^3^ UWA Oceans Institute and School of Biological Sciences University of Western Australia Perth Western Australia Australia; ^4^ Centre for Marine Science and Innovation, School of Biological, Earth and Environmental Sciences UNSW Sydney Sydney New South Wales Australia; ^5^ Institute for Marine and Antarctic Studies (IMAS) University of Tasmania (UTAS) Tasmania Australia

**Keywords:** adaptation, climate change, fucoids, future‐proofing, marine heatwave, seaweed forests

## Abstract

Marine heatwaves (MHWs) are among the most important threats to forest‐forming seaweeds and the ecological functions and services they underpin. Responses of seaweeds to MHWs are likely to vary across life‐stages, along their distribution and among populations with differing genetic structure. The effects of MHWs on early life‐stages and genetically distinct populations have rarely been tested experimentally. We used germlings and juveniles of the forest‐forming fucoid *Phyllospora comosa* from four genetically distinct populations, two at the warm‐edge (31° S) and two at the centre of its latitudinal distribution (34° S) in eastern Australia to examine their responses (i.e., survival, growth, photosynthetic efficiency, condition) to MHWs. Two MHW scenarios were tested: strong (23°C–23.5°C) and extreme (26°C), which would occur ~100 days and ~50 days per year, respectively, by 2100 under the RCP4.5 scenario (SSP2‐4.5). Survival under the extreme MHW was generally low for all populations and life‐stages (< 20% for juveniles; 20%–30% for germlings). The extreme MHW also negatively affected growth, photosynthesis and condition (e.g., loss of structural integrity in juveniles) over time. In contrast, responses of germlings and juveniles to the strong MHW did not differ from the control for either population. However, germlings from the warm‐edge population grew longer under the extreme and strong MHWs than those from the central population. Our results indicate only minor differences in responses to MHWs between genetically distinct populations of *Phyllospora*. While some *Phyllospora* individuals within each population appear able to resist strong MHWs, they are likely to be significantly affected by extreme MHWs that will be more common in the future. Given observed variation in responses among individuals within populations, finding, selecting, breeding and introducing resistant individuals (i.e., survivors to the extreme MHW) may allow increasing the resilience of *Phyllospora* forests into the future.

## Graphical abstract

Marine heatwaves (MHWs) pose a threat to global marine forests, with the effects of warming potentially being even greater on early life stages, which are often understudied. We evaluated the responses of *Phyllospora* *comosa*—one of the most crucial forest‐forming seaweeds in South‐eastern Australia—early life stages from warm‐edge and central populations to strong and extreme MHW scenarios. No effects of the strong MHW were observed, whilst high mortality occurred under the extreme scenario for both distributional ranges. Still, survivors were found after the exposure to MHWs, showing the capacity of some individuals to resist higher temperatures. Under current scenarios, *Phyllospora* might resist more common strong MHWs, but identifying traits that contribute to survival will be essential to increase the resilience of this species as extreme MHWs become more prevalent.

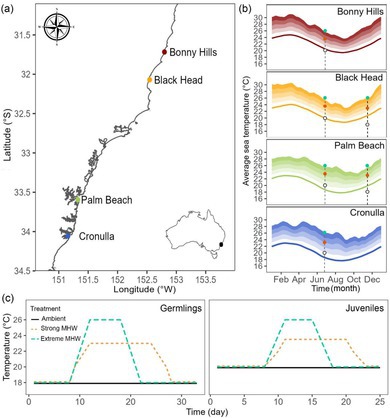

## 1 Introduction

Climate change is generating a series of changes in the environment, affecting biodiversity and degrading habitats globally (Doney et al. 2012; Harley 2011; He and Silliman 2019). In marine systems, ocean warming and marine heatwaves (MHWs) are key climate drivers causing declines of habitat‐forming species such as reef‐building corals and forest‐forming seaweeds around the world (Hoey et al. 2016; Smale 2020). MHWs are defined as abnormal temperature increases for more than 5 days above the 90th percentile threshold of the climatology (i.e., average sea surface temperature for a historical baseline) (Hobday et al. 2016) and can range from moderate to extreme (Hobday et al. 2018). MHWs are expected to increase in intensity and frequency over the next decades, with 70% of MHW events projected to be extreme (i.e., a MHW surpassing four times the temperature of the highest 90th percentile of the climatology threshold) under the RCP8.5 (SSP5‐8.5) scenario by 2100 (Oliver et al. 2019). Extreme heatwaves in terrestrial and marine environments have already generated diebacks and population disappearance for many species (Doherty et al. 2025; Filbee‐Dexter et al. 2020; Lloret and Batllori 2021; Margalef‐Marrase et al. 2020; Wernberg, Bennett, et al. 2016). There is therefore a need to increase our understanding of how habitat‐forming species will respond to MHWs and to help develop management strategies that may facilitate adaptation to higher temperatures and more frequent extreme events (e.g., assisted gene flow, Coleman et al. 2020; Wood et al. 2019, 2024).

Large‐brown seaweed forests are among the most productive and diverse habitats on coastal temperate rocky reefs and shores (Wernberg et al. 2019). These forests are composed of canopy‐forming brown algae from the orders Laminariales and Fucales (Schiel and Foster 2006) that generate a complex biogenic habitat for a wide variety of marine organisms and underpin important ecosystem functions and services (Eger et al. 2023; Smale et al. 2013; Steneck et al. 2002; Teagle et al. 2017). Seaweed forests are declining rapidly due to multiple stressors, especially warming (Smale 2020), and are expected to continue to decline (Wernberg et al. 2019). A high percentage of seaweed species have populations living close to their upper thermal limits (Laeseke et al. 2024). MHWs have driven declines in habitat‐forming seaweed populations in the southern Pacific (Thomsen et al. 2019; Wernberg, Bennett, et al. 2016) and the northern Atlantic (Filbee‐Dexter et al. 2020). For instance, after an extreme MHW in 2011, there was a partial and full disappearance of forests formed by *Ecklonia radiata* and *Scytothalia dorycarpa*, respectively (Smale and Wernberg 2013; Wernberg, Bennett, et al. 2016) in Western Australia. This resulted in a regime shift where large‐brown seaweeds have been largely replaced by turf algae (Wernberg, Bennett, et al. 2016), which cannot fulfil the ecological role and provide the same ecosystem services that seaweed forests do.

The heat stress (i.e., temperatures above the thermal optimum of species) introduced by MHWs has the potential to negatively affect physiological processes, reproduction, development and survival of marine organisms (Eggert 2012; Schwoerbel et al. 2024; Smith et al. 2023). In seaweeds, physiological traits, such as nutrient content (e.g., C:N ratio), pigment content and membrane fatty acid structure, can be altered by exposure to acute warming (Britton et al. 2020; Flukes et al. 2015; Wernberg, de Bettignies, et al. 2016). Some of these traits, such as increased membrane fluidity (Britton et al. 2020; Los and Murata 2004) and the expression of heat shock proteins (Jueterbock et al. 2014; Smolina et al. 2016), can help improve the responses to stress. These responses can vary between species within the same taxonomic group (e.g., phaeophyceans and marine angiosperms; Bennett et al. 2022, or cnidarians; Bahr et al. 2018), but also between individuals and populations of the same species. For example, warm‐edge seaweed populations may have higher thermal resistance limits (Bennett et al. 2022, 2015; Ferreira et al. 2014; Harris 2024; King et al. 2019). Such increased thermal resistance is likely due to local adaptation as a response to constant environmental pressure (Bennett et al. 2015), a trait that may make some seaweeds more resistant to extreme events, such as MHWs. However, other seaweeds from warm‐edge populations may be less resilient, with even narrower limits for optimal performance temperatures (Clark et al. 2020, 2018; Hernández et al. 2023; Pearson et al. 2009).

Experimental assessment of differences in MHW responses between populations is essential to determine the vulnerability of species to extreme events. However, most of the studies that evaluate thermal responses between populations focus on adults (Bennett et al. 2022, 2015; Mota et al. 2018; Pearson et al. 2009) rather than early life‐stages (but see Clark et al. 2020), which are often understudied (Edwards 2022; but see Schwoerbel et al. 2024). Early life‐stages are often more sensitive to warming and other stressors than adults (Al‐Janabi et al. 2016; Fredersdorf et al. 2009; Nielsen et al. 2014), potentially enhancing the negative impacts of MHWs on their physiology. Understanding the responses of early life‐stages can provide a more holistic view of the population responses to MHWs, as they are the base for recovery after mass mortality, and are therefore key for the sustainability of seaweed forest populations (Edwards 2022; Lotze et al. 2001).

*Phyllospora comosa* (Labillardière) C.Agardh (hereafter *Phyllospora*) is a dominant forest‐forming species on shallow rocky reefs in south‐eastern Australia (Coleman and Wernberg 2017; Underwood et al. 1991) that hosts a wide variety of organisms, including economically valuable species such as abalone and crayfish (Andrew 1999; Marzinelli et al. 2016, 2014). *Phyllospora* is distributed from Port Macquarie, in New South Wales (31°35′39.7S 15″2°50′36.4″ E) and extends along the eastern and southeastern Australian coastline and around Tasmania (Coleman and Wernberg 2017). *Phyllospora* is particularly sensitive to warming (Young et al. 2023) and is expected to decline significantly, potentially becoming extinct by 2100 under the RCP6.0 (SSP2‐4.5—SSP3‐7.0) scenario (Martínez et al. 2018). Whilst a limited number of experiments testing heat stress‐related responses have been done to test the sensitivity of this species, these have focused on populations outside the warm edge of *Phyllospora's* range (Britton et al. 2020; Flukes et al. 2015; Straub et al. 2022). However, populations of *Phyllospora* exhibit distinct genetic structures across their warm, central and leading‐edge ranges, including at *loci* associated with sea surface temperature (Wood et al. 2021). These differences suggest potential genetic variation in thermal tolerance, with warm‐edge populations potentially more resilient to higher temperatures (Wood et al. 2021).

In this study, we aimed to determine if there are any differences in tolerance to MHWs between warm‐edge and central populations of *Phyllospora* using two early life‐stages: germlings (1 week old) and juveniles (> 6 months old). Each life‐stage was exposed to two MHW profiles: one representing a strong MHW event, where the highest temperature surpassed the upper 90th percentile limit of the species temperature threshold two times (based on average temperatures from 1982 to 2011), and one representing an extreme MHW event, corresponding to maximum temperatures surpassing the climatology threshold upper limit four times. These events are expected to occur ~100 (strong MHW) and ~50 days (extreme MHW) per year by 2100, when 50% of the ocean would be in a permanent MHW state under the RCP4.5 scenario (SSP2‐4.5) (Oliver et al. 2019). Given the known differences in genetic structure and exposure to different temperatures between warm‐edge and central populations (Wood et al. 2021), we hypothesised that warm‐edge populations, which are exposed to higher temperatures, would be more resilient to MHWs. We predicted that MHWs would have a greater negative effect on the survival, condition (e.g., slower growth, greater bleaching and loss of structural integrity) and physiology (e.g., lower rates of photosynthesis, lower pigment content and lower C:N ratio) of juveniles and germlings from central populations than on those from warm‐edge populations. If responses depend on the severity of the MHW, we hypothesised that the extreme MHW would generate a greater negative impact on these responses (i.e., lower survival, growth, photosynthesis and worse condition) than the strong MHW.

## 2 Materials and Methods

### 2.1 Study Sites

**FIGURE 1:**
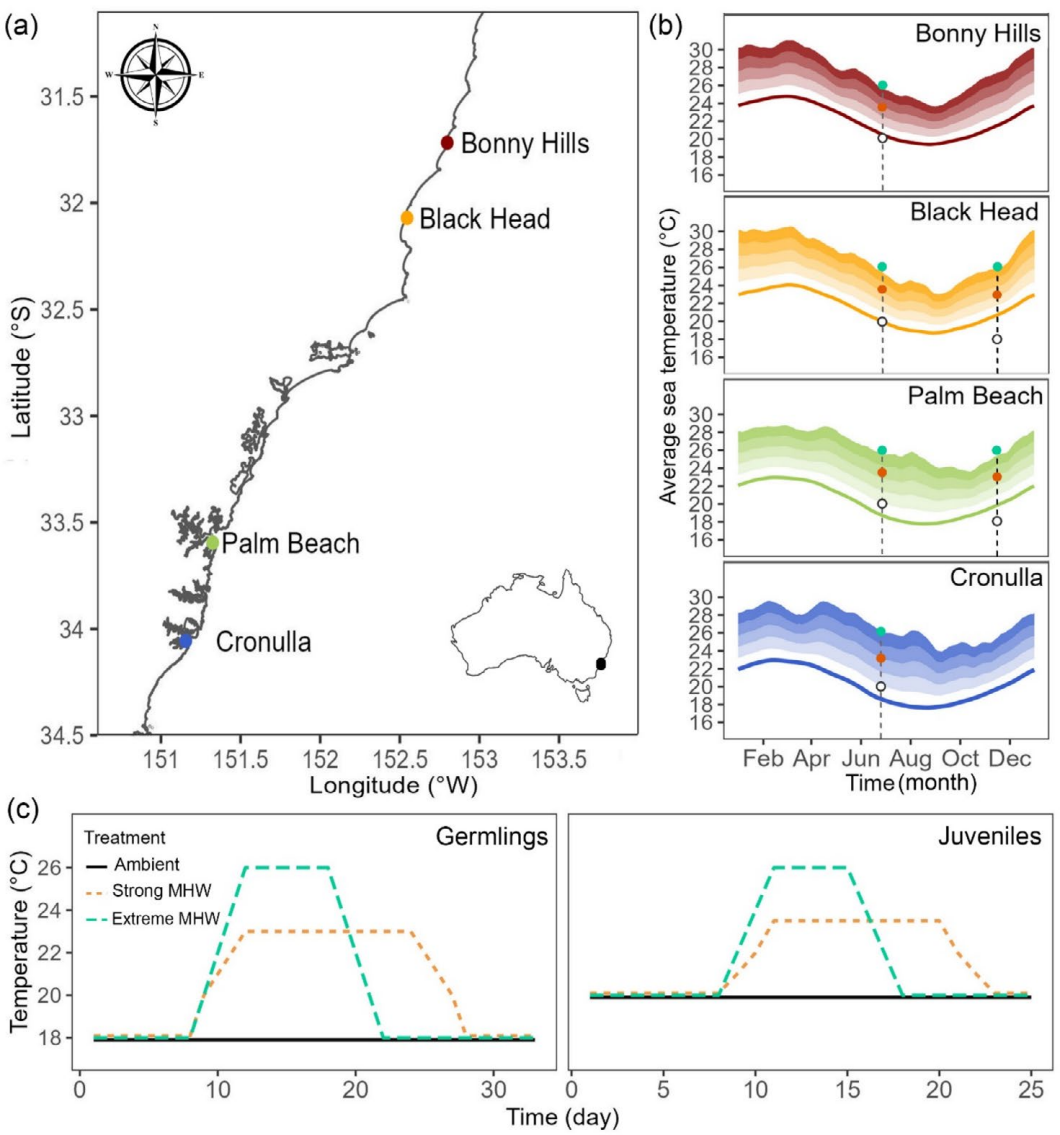
(a) Study sites along the east coast of NSW where *Phyllospora comosa* juveniles and blades (to generate germlings) were collected. (b) Long‐term average temperature (1982–2011) climatology and Marine Heatwave (MHW) thresholds for each site. Thresholds are represented by shaded areas from lighter to darker colour, corresponding to a moderate, strong, severe and extreme MHW, respectively, according to Hobday et al. (2018) definition. Data for average temperature and thresholds were obtained from the Marine Heatwave Tracker climatology and thresholds database (https://www.marineheatwaves.org/), utilising the nearest coordinates to each study site. Coloured dots indicate the maximum temperature used in experimental MHWs, with turquoise corresponding to the extreme MHW, orange to the strong MHW and white to the ambient used in each experiment. Dots are positioned in the corresponding collection time (dashed vertical lines) for each experiment, with germlings being collected in November 2022 and juveniles collected in June 2023; and (c) MHW profiles used for germling and juvenile experiments.

*Phyllospora* juveniles and reproductive blades were collected from two warm‐edge populations: Bonny Hills (31°35′39.7″S 152°50′36.5″ E) and Black Head (32°04′11.8″S 152°32′45.7″ E) and two central populations: Palm Beach (33°35′58.1″S 151°19′41.0″ E) and Cronulla (34°04′13.7″S 151°09′24.8″ E) in New South Wales, eastern Australia (Figure 1a). The genetic structure of Bonny Hills and Black Head (warm‐edge populations) has been previously observed to differ from that of Palm Beach and Cronulla (central populations) (Wood et al. 2021). These sites are exposed to different upper and lower temperature limits throughout the year, with Bonny Hills normally reaching the highest temperatures (24.8°C in February compared to 22.9°C in Cronulla) and Cronulla the lowest (17.7°C in August, compared to 19.4°C in Bonny Hills, Figure 1b). Records indicate that these populations have been exposed to a total of 143 strong and 22 severe MHWs since 1982 (some shared events), with 34 events occurring in Bonny Hills, 38 in Black Head, 47 in Palm Beach and 44 in Cronulla (Table S1). Climatology and MHW data are based on satellite measurements of Sea Surface Temperature (SST), which can be different from coastal values to which *Phyllospora* is exposed in each site; however, they indicate baselines and long‐term patterns of temperature variations among sites (lowest and highest values), being often correlated to data recorded in situ (Bernardello et al. 2016; Smale and Wernberg 2009; Stobart et al. 2015).

### 2.2 Experiments With Germlings

#### 2.2.1 Collection and Processing of Reproductive Blades

Forty pieces of *Phyllospora* reproductive blade tissue (10–15 cm length) were collected on November 9, 2022, from each study population. Blades were collected from individuals separated by at least 1 m, snorkelling parallel to the coastline at ~0.5–3 m depth to avoid sampling highly related individuals. Male and female blades were sorted by visual differences in their conceptacles (rounded for females and elongated for males; Figure S1), stored in separate black bags, and transferred into a cold esky (temperature maintained with icepacks) to avoid early spawning. The collected material was transported by car to the Sydney Institute of Marine Science (SIMS) located in Chowder Bay (33°50′18.0″S 151°15′23.6″ E) to be processed on the same day. All reproductive blades were processed for spawning 7–9 h after collection.

Spawning and in vitro fertilisation were carried out by adapting the method used by Cumming et al. (2019) using 15 female and 15 male blades. Four 3.5 cm long blade segments (starting from the base of the blade, 4–6 mm width) from each female (60 blades in total) were rinsed with 1 μm filtered seawater, dried and placed in a 500 mL glass beaker with 300 mL of 1 μm filtered seawater. Ten male blade sections for each male were rinsed with 1 μm filtered, dried and placed into individual 60 mL containers, where 10 mL of 1 μm filtered seawater were added and left for 40–60 min to spawn. Once the sperm and eggs were released, sperm were pooled together and 15 mL of the sperm mixture were added to the female blade container. After 24 h, the remaining female blades were removed from the container, and similar densities of fertilised eggs (~50 per well) were moved into 6 x culture well plates, with three wells in the same plate for each population. The same process was carried out for the four populations on the same day in a temperature‐controlled room at 18°C. The blades collected earlier were the first to be processed. Although fertilisation was attempted in all four populations, viable germlings were only obtained for one warm‐edge (Black Head) and one central population (Palm Beach). Cultures were kept in a 12:12 light:dark cycle with a light intensity of ~50 μmol photons m^−2^ s^−1^ (PAR) and the filtered seawater was changed twice per week.

#### 2.2.2 MHW Experiments

Eight days after fertilisation, germlings were exposed to two different MHW scenarios based on the definition of Hobday et al. (2018) and climatology during the collection dates (Figure 1c). The MHW profiles represented a strong and an extreme MHW. Intensity levels were defined as the number of times the highest temperature of a MHW surpasses the 90th percentile threshold of the climatology of a site (1×, 2×, 3×, 4×, based on average site temperatures), with the strong MHW exceeding the climatology threshold by twice the highest temperature and the extreme MHW four times. The strong MHW lasted 20 days, ranging from 18°C to 23°C. Temperature started increasing between days 8 and 9 (18°C—20°C) and then by 1°C per day until the MHW peak on day 12 (23°C). On day 28, the temperature began to decrease at the same rate as it had previously increased. The extreme MHW lasted 14 days, starting at 18°C and reaching 26°C. The temperature increase was steeper with a rise of 2°C per day from day 9 to day 12. The temperature decreased at the same rate from day 25 to day 28. The MHW profiles had different durations to maintain a similar absolute heat pressure between MHW treatments: 83°C (strong MHW) and 80°C (extreme MHW). These values only consider the excessive temperature (area between the ambient and MHW lines). Ambient temperature was 18°C due to previous success in spawning and germling growth under this temperature, and the seawater temperature ranged between 18°C and 19°C during the time of collection (early November 2022).

Water baths were used to control temperature changes, with six randomly placed water baths for each temperature treatment (Figure S2). Each water bath had two well plates inside (6 wells × 4.5 mL each) where the germlings settled.

#### 2.2.3 Survival and Growth

The survival, length (mm) and area (mm^2^) of the germlings were quantified to evaluate their responses to simulated MHWs. The survival of germlings was quantified twice per week, starting from day 9 until day 30, by counting the number of live individuals in each well at each time point. An individual was classified as ‘live’ when the structure was maintained (i.e., no cellular content being expelled) and yellow‐brown colouration was observed (e.g., Figure S3). Photos of the germlings (1–4 individuals per well) were taken every 7 days (days 9–30) to measure their length and area and describe their development. The length (mm) and area (mm^2^) of the germlings were measured considering the body of the individual without the rhizoid (Figure S3) using the software ImageJ (Schneider et al. 2012). The rhizoid was excluded from the measurements as its length observed in the photos would depend highly on the perspective and the attaching position. Individuals were also described using three morphological categories: elongated, branched or rounded (Figure S4).

#### 2.2.4 Statistical Analyses

Differences in survival, length (mm) and area (mm^2^) and morphological category (proportion elongated, branched or rounded; averaged per water bath; *n* = 6) among Populations (fixed, 2 levels = Black Head and Palm Beach), MHW (fixed, crossed, 3 levels = Ambient, strong MHW and extreme MHW) and Day (fixed, crossed, 4 levels = 9 (start of the MHWs), 16 (during MHWs), 23 (after extreme MHW), 30 (after strong MHW)) were analysed using linear mixed models (LMMs) with water bath as a random factor to account for potential non‐independence due to repeated sampling of the baths over time. Each water bath was considered as a replicate (*n* = 6).

Model assumptions were checked by inspecting plots of residuals versus fitted values (Zuur et al. 2010). Significance was assessed using the ‘Anova’ function in the R *car* package (Fox et al. 2001). Tukey post hoc contrasts were carried out using the function ‘emmeans’ package *emmeans* (Lenth 2017) to observe significant main effects when significant interactions occurred. All analyses were carried out in R Studio 2024.12.0 (R Core team 2024).

### 2.3 Experiments With Juveniles

#### 2.3.1 Juvenile Collection

25–35 *Phyllospora* juveniles (10–20 cm) were collected from the same four populations (~0.5–3 m depth) as the germlings (Figure 1a) on June 15–16, 2023. They were haphazardly collected via snorkelling (> 1 m apart) by gently detaching their holdfast from the bottom. After collection, juveniles were stored in plastic bags and kept in cool (~4°C–10°C), dark conditions and transported to the aquarium of SIMS and transferred to 2 L individual containers with UV‐filtered seawater at 20°C. On the same day, a photo of each individual was taken to quantify initial length (whole individual, Figure S5) and status. Juveniles were left for 8 days in individual containers at 20°C to acclimate before starting the MHW experiments. The ambient temperature was 20°C (± 0.5°C), similar to the temperatures on the collection date in all populations (19°C–20°C). The water in individual containers was changed every 2 days and disposed of to avoid mixing between containers. Juveniles were under a 12:12 light:dark cycle with a light intensity of ~50 μmol photons m^−2^ s^−1^ (PAR).

#### 2.3.2 MHW Experiments

Individual 2 L containers were placed into six replicate water baths for each temperature treatment (Figure S6). One or two randomly selected individuals per population were placed in the same water bath. Water baths were located on top of shelves (three water baths per shelf, one for each MHW treatment) that were under a different lighting source (maintaining light intensity of ~50 μmol photons m^−2^ s^−1^). There were 8–12 individuals for each treatment combination (population and temperature) at the start of the experiment.

Juveniles were exposed to two different MHWs (Figure 1c, Figure S6). The strong MHW increased from 20°C to 23.5°C and lasted a total of 15 days, exerting an absolute warming during the experiment of 41°C (area under the curve). The temperature started increasing on day 9 by 1°C per day (1.5°C from day 10 to 11). The extreme MHW lasted 10 days, ranging from 20°C to 26°C, with an absolute warming of 42°C. The temperature increase (day 9 to 11) was faster than the strong MHW, with 2°C per day. The duration of the juvenile MHWs was shorter due to the general decay of juveniles under aquarium conditions in previous trials. It is important to note that germling MHW profiles therefore represent a higher temperature increase and heat pressure than juvenile MHWs. Although this prevents us from making direct comparisons between the two life‐stages, we are still able to evaluate the broader responses of *Phyllospora*'s early life‐stages to warming.

#### 2.3.3 Survival and Condition

To test how juveniles from different populations responded to warming, we quantified their survival, condition (bleaching, fouling, necrosis, green colouration, structural integrity loss and Fv/Fm) and relative growth. We quantified survival, bleaching, fouling, necrosis, green colouration and structural integrity loss (Figure S7) at the start of the acclimation period and every 2 days following the start of the MHW (*n* = 8–12). Survival was measured by giving a value to dead or live individuals (1 = ‘live’, 0 = ‘dead’). Bleaching was identified by the presence of decoloured/white spots. Fouling was defined as the amount of other organisms (e.g., seaweed) covering the juvenile tissue. Necrosis was defined as the presence of dark tissue (occurring especially in the blade edges). Green colouration occurred when the blade pigment turned green (mostly visible underwater). Structural integrity loss refers to the tissue disintegration (Figure S7). These measurements were given a score (0–4) corresponding to 0%, < 25%, 30%–40%, 60%–70% and > 80% of the juvenile tissue with the condition.

Photosynthetic quantum yield (Fv/Fm; *n* = 8–12) of PSII was measured to estimate the ‘photosynthetic health’ (Hurd et al. 2014) using a diving pulse‐amplitude‐modulated fluorometer (Diving PAM, Waltz) on days 8 (start of the MHW), 12 (highest temperature), 18 (end of the extreme MHW) and 22 (end of the strong MHW). This methodology was chosen as it can be an efficient method to estimate general stress in seaweeds, including *P. comosa* (e.g., Straub et al. 2022; Flukes et al. 2015); however, it may not account for mild or transient stress, which requires additional measurements such as oxygen consumption and release (Logan et al. 2007). Before any measurement (measuring light intensity = 5 Hz, saturation intensity = 8 μmol m^−2^ s^−1^, saturation pulse duration = 0.8 s), individuals were acclimated for 10 min in full dark conditions.

The total length of the plants was measured on the collection day (day 0), at the beginning of the MHW (initial length at day 8) and when the experiment ended (final length at day 25, 3 days after the end of the MHW). The relative growth rate (RGR %d^−1^) was calculated based on length differences to minimise artefacts caused by manipulating individuals, modifying the equation used by Straub et al. (2022):

$$\mathrm{RGR}\left[\%d^{-1}\right]=\left[{\left(\frac{\mathrm{Lf}}{\mathrm{Li}}\right)}^{\frac{1}{t}}-1\right]\times 100$$

where Lf = final length, Li = initial length at respective day and *t* = days passed in treatment.

Additionally, at the end of the experiment, the tissue of each surviving individual (*n* = 5–12) was collected and freeze‐dried for C:N and pigment analysis. This occurred on day 25 for ambient and strong MHW treatments and on day 20 for extreme MHW (due to mortality and disintegration), both corresponding to 2 days after the end of the corresponding MHW.

#### 2.3.4 C:N Ratio and Pigments

We evaluated non‐lethal effects of MHWs on survivors by determining tissue carbon and nitrogen and pigment content (chlorophyll‐a, ‐c and fucoxanthin, mg g^−1^). Freeze‐dried samples of *Phyllospora* were ground using a ball mill Retsch MM400. Samples of approximately 2 mg were weighed in tin capsules. Contents of carbon and nitrogen were measured with an elemental analyser (Elementar Vario Isotope Cube) with helium as a carrier gas and combustion at 950°C and reduction at 600°C. Samples devoid of water were obtained using phosphorus pentoxide. This analysis was done at the Flinders Analytical Laboratory using six randomly collected replicates from the different populations and MHW treatments.

For pigment extractions (chlorophyll‐a, ‐c and fucoxanthin), we added 4 mL of dimethyl sulfoxide (DMSO) to a 10 mL tube with 0.12–0.15 g of freeze‐dried *Phyllospora* blade pieces. Blades were previously cut into pieces to facilitate extraction (Smart et al. 2022). After 10 min, the liquid was collected in another tube and stored until measured. For the second extraction, we added 6 mL of 90% acetone (v/v), which was left for 30 min. After the extraction time, the liquid was collected in another test tube. Using a double beam spectrophotometer (VWR, UV‐6300 PC), we measured the absorbance of the DMSO and acetone extracts to calculate the pigment content (following equations by Seely et al. 1972). The measurements were conducted at wavelengths of 665 (chlorophyll‐a, ‐c, fucoxanthin), 631 (chlorophyll‐c, fucoxanthin), 581 (chlorophyll‐c, fucoxanthin) and 470 (fucoxanthin) for DMSO extracts, and 664 (chlorophyll‐a, ‐c, fucoxanthin), 631 (chlorophyll‐c, fucoxanthin), 581 (chlorophyll‐c, fucoxanthin), 470 (fucoxanthin) for acetone extracts. Extracts were analysed immediately after being collected. 6–9 random replicates were processed for each population and MHW due to tissue availability.

#### 2.3.5 Statistical Analyses

Differences in condition (including bleaching, fouling, necrosis, structural integrity loss, Fv/Fm; *n* = 8–12) among Populations (fixed, 4 levels: Bonny Hills, Black Head, Palm Beach and Cronulla) and MHW (fixed, crossed, 3 levels: Ambient, strong MHW, extreme MHW) were tested separately for Days 8 (start of the MHWs), 12 (during MHWs), 18 (end of extreme MHW) and 22 (end of strong MHW) using linear models (LMs). Individual analyses were performed for each day separately, as data from the extreme MHW on day 22 were not available due to high mortality.

To evaluate differences in survival, we ran generalised linear models (GLMs) with binomial distribution (1 = ‘live’, 0 = ‘dead’) using MHW treatment and population as fixed factors, as described above. Each individual was considered as a replicate (*n* = 8–12). Significant differences in survival at days 8 and 12 were not tested due to almost no mortality observed.

Relative growth rate (*n* = 8–12) and final C:N ratio (including tissue carbon and nitrogen; *n* = 6–5) and pigments (chlorophyll‐a, ‐c and fucoxanthin; *n* = 5–9) were contrasted between populations (fixed, 4 levels: Bonny Hills, Black Head, Palm Beach and Cronulla) and MHW scenario (fixed, crossed, 2 levels: Ambient and strong MHW) using LMs. Only ambient and the strong MHW were contrasted for these analyses due to the high mortality during the extreme MHW.

For LMs and GLMs, significance (alpha = 0.05) was assessed using the ‘Anova’ function in the R *car* package (Fox et al. 2001). Tukey post hoc contrasts were tested using the function ‘emmeans’ from the package *emmeans* (Lenth 2017) to explore significant main effects of significant interactions. Model assumptions were checked as above, by inspecting plots of residuals vs. fitted values (Zuur et al. 2010), and the data were square root transformed to meet normality assumptions.

## 3 Results

### 3.1 Germling Responses to MHWs

**FIGURE 2:**
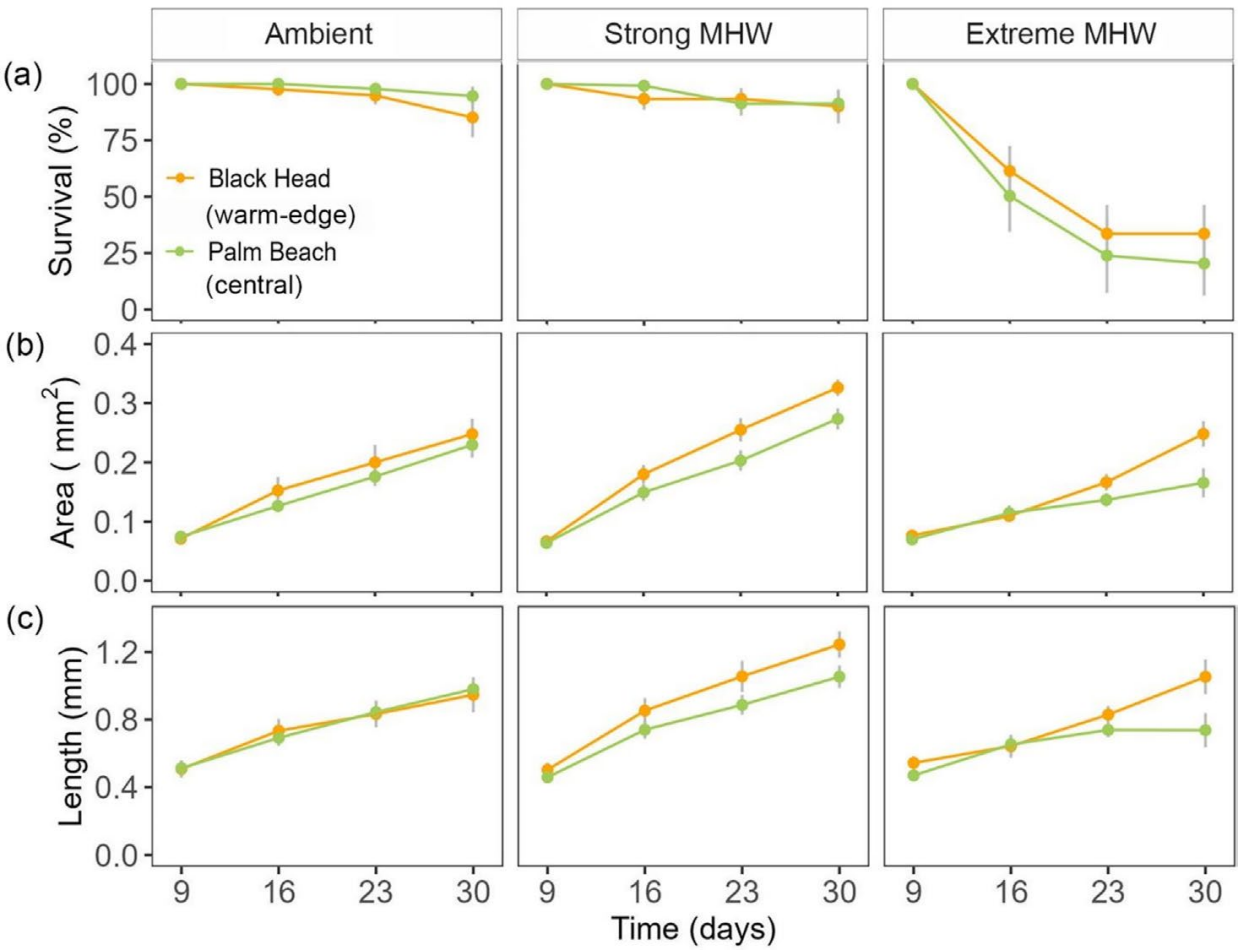
*Phyllospora comosa* germling (a) survival, (b) area (mm^2^) and (c) length (mm) (mean, ± SE; *n* = 6) under different MHW scenarios. Germlings were obtained by fertilising gametes from Black Head and Palm Beach. Maximum temperature of the strong MHW was 23°C, and 26°C for the extreme MHW. Ambient treatment was maintained at 18°C as a control.

Germling survival did not differ among populations (*χ* ^2^ = 0.22, df = 1, *p* = 0.64; Table S2 for mean values). However, differences were observed between MHW treatments on some days (*χ* ^2^ = 98.27, df = 6, *p* < 0.001; Table S3; Figure 2a). No significant mortality occurred under ambient or strong MHW (Figure 2a, Table S3). However, germlings under the extreme MHW showed lower survival than the other MHW treatments from day 16, which decreased further over the experiment. Only 33% of the individuals from Black Head and 20% in Palm Beach survived by the end of the experiment (Figure 2a).

Germling area increased over time across all populations and MHW treatments (Figure 2b). Differences between populations occurred from day 23 onwards, independent of the MHW treatment (*χ* ^2^ = 13.8, df = 3, *p* < 0.01; Table S4), with Black Head individuals having significantly greater surface area than those from Palm Beach. An interaction was also found between MHW treatment and day (*χ* ^2^ = 51, df = 6, *p* < 0.001). Individuals exposed to the strong MHW were consistently larger in area than those under an extreme MHW from day 16 onwards, and than those under ambient conditions from day 23 (Table S4; Figure 2b). Length followed similar trends to area (Figure 2c). It was influenced by an interaction between population and MHW treatment, regardless of day (*χ* ^2^ = 13.16, df = 2, *p* < 0.01). Black Head germlings were significantly longer than Palm Beach germlings under both the strong MHW and the extreme MHW conditions (Table S5). Differences among the strong, extreme and ambient treatments emerged by day 16 (*χ* ^2^ = 29.23, df = 6, *p* < 0.001), with germlings under the strong MHW being consistently larger than the extreme MHW from day 16, and than ambient on day 30 (Table S5).

Germlings across all populations and MHW treatments initially displayed an elongated shape (Figure S8), which remained largely unchanged over the 30 days of the experiment (*χ* ^2^ = 7.01, df = 3, *p* = 0.07, Table S6). Branching began after day 16, leading to a significant increase in the number of individuals with a branched morphology over time (*χ* ^2^ = 41.14, df = 3, *p* < 0.001). However, the proportion of branched individuals did not differ significantly between MHW treatments or populations (Table S7). Some germlings also exhibited a rounded shape at the start of the experiment (Figure S8). Although no differences were observed between MHW treatments throughout the 30 days (*χ* ^2^ = 0.55, df = 2, *p* = 0.56), this shape differed between populations being more frequent in germlings from Black Head than Palm Beach (*χ* ^2^ = 5.68, df = 1, *p* = 0.02, Table S8). This morphology also became less common over time for both populations (*χ* ^2^ = 5.68, df = 1, *p* = 0.02).

### 3.2 Juvenile Responses to MHWs

**FIGURE 3:**
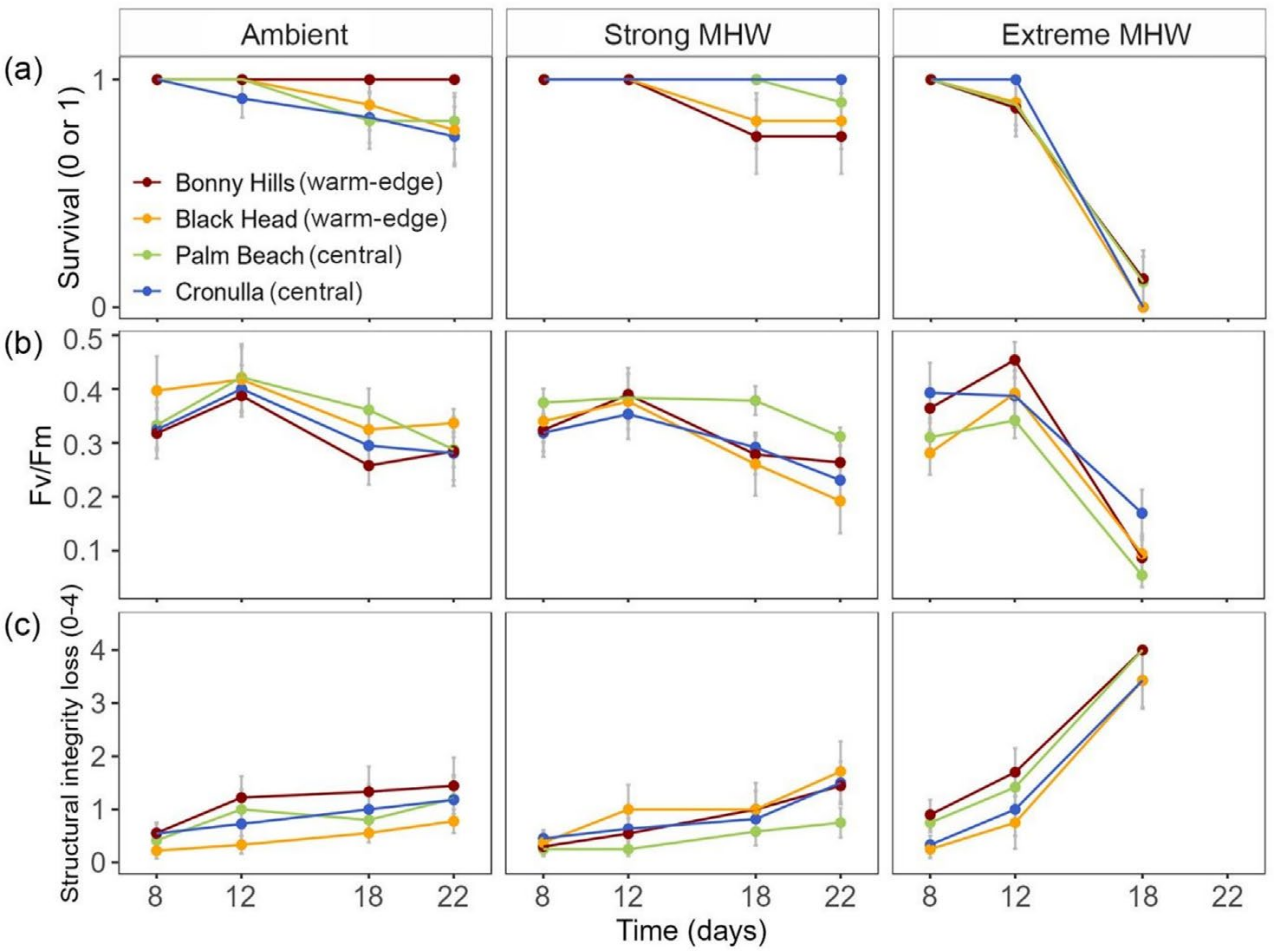
*Phyllospora comosa* juvenile survival, photosynthetic efficiency (Fv/Fm) and structural integrity loss (mean +\− SE; *n* = 8–12) across populations and MHW scenarios. Strong marine heatwave reached 23.5°C, and extreme MHW reached 26°C. Ambient treatment was maintained at 20°C as a control. Survival was recorded as either ‘live’ (1) or ‘dead’ (0) individuals.

Survival of juveniles remained close to 100% during the initial days of the MHW treatments (days 8 and 12; Table S9; Figure 3a). No differences in survival were observed between populations at any point of the experiment (*χ* ^2^ = 0.42, df = 3, *p* = 0.94). However, significant differences between MHW treatments were found on day 18 (*χ* ^2^= 89.41, df = 2, *p* < 0.001; regardless of population) when mortality was significantly higher under the extreme MHW than under the strong MHW or ambient conditions (Table S10), with over 80% mortality in the extreme treatment. Following the removal of individuals under the extreme MHW prior to the end of the experiment (due to the disintegration of dead individuals), no differences in survival were observed between ambient and strong MHW treatments or among populations by day 22.

Juvenile length, measured as relative growth rate (RGR, % d^−1^), declined over time, with negative RGR values (Figure S9; Table S11) and did not differ among populations (*F*_3,20_ = 0.75, *p* = 0.53) or MHW treatment (*F*_1,20_ = 0.1, *p* = 0.76). Photosynthetic efficiency (Fv/Fm) did not differ among populations throughout the experiment (Figure 3b; Table S12). However, differences between MHW treatments were observed on day 18 (*F*_2,103_ = 44.7, *p* < 0.001), when Fv/Fm was significantly lower under the extreme MHW compared to strong MHW and ambient treatment across all populations.

Differences in the average loss of structural integrity among populations were not observed during the experiment (Figure 3c; Table S13). However, individuals showed significantly different tissue structural integrity loss across MHW treatments from day 12 (*F*_2,109_ = 3.9, *p* = 0.02), with the extreme MHW resulting in greater structural loss than the other MHW treatments. This tissue deterioration increased by day 18 (*F*_2,95_ = 67.86, *p* < 0.001). The amount of fouled tissue also did not differ among populations throughout the experiment (Figure S10; Table S14). However, it was significantly lower under the extreme MHW than ambient on day 18 (*F*_2,95_ = 4.96, *p* < 0.01), regardless of the population. No differences were observed in bleached tissue from day 8 to day 18 (Table S15). However, a significant interaction between population and MHW treatment occurred on day 22, with Cronulla juveniles under the strong MHW showing more bleaching in their blades than under ambient conditions (*F*_3,69_ = 2.87, *p* = 0.04). The amount of necrotic tissue in juveniles was higher in germlings from Palm Beach than Cronulla (both central populations) in all MHW treatments, but only on day 18 (*F*_3,95_ = 2.92, *p* = 0.04; Table S16). Necrosis in juveniles was also elevated under the extreme MHW on day 12, regardless of population (*F*_2,110_ = 3.27, *p* = 0.04). Differences in the amount of tissue with green colouration occurred with an interaction between MHW treatment and population on days 8 (*F*_6,109_ = 3.44, *p* < 0.01) and 12 (*F*_6,110_ = 3.52, p < 0.01). Green colouration in blades was higher in Black Head under the extreme MHW compared to all other populations and MHW treatments on both days (Table S17). On day 18, differences occurred between MHW treatment, regardless of population (*F*_2,95_ = 7.7, *p* < 0.001), with individuals having the most green colouration in blades under the extreme MHW.

**FIGURE 4:**
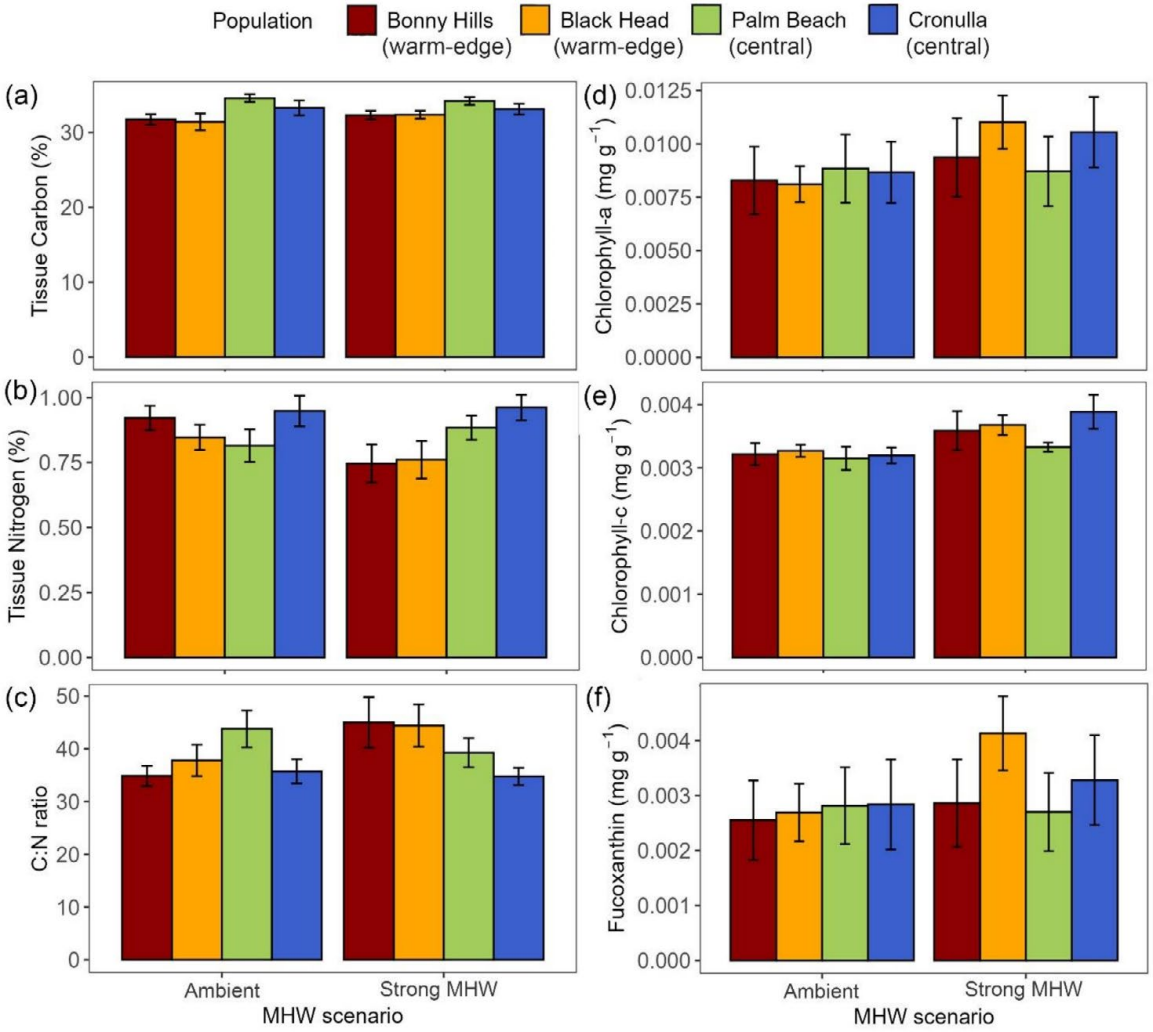
Tissue (a) carbon, (b) nitrogen (%) and (c) C:N ratio (mean +\− SE; *n* = 5–6) and (d) chlorophyll‐a, (e) ‐c and (f) fucoxanthin content (mg g^−1^; mean +\− SE; *n* = 5–9) in *Phyllospora comosa* juveniles from four populations along NSW exposed to a strong MHW (max. temp. = 23.5°C) and ambient conditions (20°C).

Tissue carbon content (%) differed among populations for all MHW treatments (*F*_3,38_ = 4.77, *p* < 0.01), with Palm Beach juveniles having higher carbon content than those from Black Head and Bonny Hills (Figure 4a; Table S18). No differences were observed in tissue nitrogen and C:N ratio between population or MHW (Figure 4b,c; Table S18). Chlorophyll‐a and fucoxanthin levels did not differ among populations and MHW treatments (Figure 4d,f, Table S19). However, chlorophyll‐c differed among MHW treatments, regardless of population (*F*_1,47_ = 9.32, *p* < 0.01; Figure 4e), being significantly higher under the strong MHW than ambient conditions.

## 4 Discussion

Ocean warming and MHWs threaten the persistence of seaweed forests, but impacts are likely to vary among individuals and populations exposed to differing environmental conditions (Bennett et al. 2015; Starko et al. 2024). Here, we found similar responses from genetically distinct warm‐edge and central populations of *Phyllospora* to simulated MHW events. Regardless of population, no negative impacts occurred after exposure to a simulated strong MHW. However, high mortality and sublethal impacts such as loss of structural integrity and reduced photosynthetic efficiency (Fv/Fm) were observed for all populations under an extreme MHW scenario. Notably, a few individuals from each of the populations sampled were able to survive even under extreme conditions, indicating that some mechanisms of resilience may exist in *Phyllospora* populations.

We found no evidence of enhanced tolerance to MHWs in warm‐edge juveniles compared to those from central populations. However, germlings from the warm‐edge increased their length/area under a strong MHW more than central populations. Previous studies have found genetic differences and potential links between some *loci* and SST in their environment (Wood et al. 2021). Upper thermal limits appear to be higher for populations from lower latitudes (Harris 2024), which might explain the larger sizes of germlings from Black Head (warm‐edge) compared with those from Palm Beach (central) under MHW treatments (especially the strong MHW, where no significant mortality occurred in any population). In that sense, *Phyllospora* warm‐edge populations may still be more resistant to thermal stress, probably tolerating better the exposure to strong MHWs in the long term. The exposure to warmer conditions may drive local adaptation in marine organisms (López‐Goldar and Agrawal 2021), which may narrow thermal ranges, but favour individuals with higher upper limits (King et al. 2019). Indeed, some studies have shown that macroalgae from warm‐edge populations can tolerate higher temperatures (Bennett et al. 2022, 2015; King et al. 2019). This situation may also occur in central populations that have historically been exposed to more MHWs, likely increasing selection and/or resistance of individuals that have already been exposed to heat stress (King et al. 2025; McCarthy et al. 2024). Still, our results indicate that under extreme events, this potential local adaptation, if present, may not be sufficient to survive.

The high levels of mortality of germlings and juveniles in response to the extreme MHW suggest major physiological impacts. The exposure to high temperatures can generate tissue necrosis and mortality, as observed in previous experimental studies on fucoid germlings (Andrews et al. 2014; Capdevila et al. 2019) and juveniles (Flukes et al. 2015; Straub et al. 2022). These findings are also consistent with field observations on other species, where entire fucoid seaweed populations disappeared in response to extreme MHWs (Smale and Wernberg 2013; Wernberg, Bennett, et al. 2016), a risk that might be even greater for early life stages, which can be more sensitive to warming (Al‐Janabi et al. 2016; Nielsen et al. 2014).

The extreme MHW temperature of 26°C used in this study exceeds the optimal values recorded for *Phyllospora* photosynthesis (Veenhof et al. 2024). Exposure to suboptimal temperatures over extended periods can generate structural damage and impair cellular processes, such as protein and enzyme stability (Eggert 2012), which may explain the decay and mortality of *Phyllospora* germlings and juveniles that started just a few days after the extreme MHW reached its peak temperature. Other studies on seaweeds have identified potential impacts of exposure to suboptimal temperatures, which may make populations at the warm edge more vulnerable to extreme events (Clark et al. 2020, 2018; Martínez et al. 2012; Pearson et al. 2009). For instance, warm‐edge populations are often found in lower densities (Clark et al. 2018) and display lower recruitment and/or lower densities than in the central range (Andrews et al. 2014). Additionally, germlings and adults of the intertidal seaweed *Hormosira banksii* (Turner) Decaisne, which shares a similar distribution to *Phyllospora* along the Eastern Australian coast, are more thermally sensitive in the warm‐edge than central populations (Clark et al. 2020, 2018). This response was attributed to restricted gene flow and inbreeding occurring in the warm‐edge populations, generating low genetic diversity (Clark et al. 2020), which may also be experienced by *Phyllospora*. There is a clear break in the genetic similarities and low diversity in *Phyllospora* populations from Black Head (31° S; FST > 0.275 and 54%–72% of the diversity in central populations) to the north, based on previous studies utilising Single‐Nucleotide Polymorphism (SNPs; Wood et al. 2021), likely making them more vulnerable to range contractions under extreme events.

It is important to note that these experiments took place during the winter and spring months, and the reduced thermal tolerance of juveniles and germlings may also be attributed to a seasonal effect, as individuals often respond worse to temperature increases while in cooler months (Al‐Janabi et al. 2016; Andrews et al. 2014). However, *Phyllospora* juveniles collected in early autumn, when temperatures are the highest (March), also showed high mortality (> 80%) under a 27°C MHW in a different study (Straub et al. 2022). This indicates a likely high vulnerability of this species to extreme MHWs throughout the year, independent of season and population, especially considering higher temperatures expected during summer MHWs.

Even with the high mortality observed under the extreme MHW treatment, ~10%–30% of germling and juvenile individuals from certain warm‐edge and central populations survived the extreme MHW (while > 85% survived in ambient conditions). Responses of individuals to stressors are likely to vary, and previous experiments with *Phyllospora* and other seaweeds have shown different individual responses to temperature stress (Alsuwaiyan et al. 2021; Andrews et al. 2014; Flukes et al. 2015; Straub et al. 2022). This is supported by high variability in thermal tolerance observed on *Phyllospora* adults from the same population along their latitudinal distribution (Harris 2024). These individuals might, for example, be characterised by specific genotypes (Alsuwaiyan et al. 2021) or inherit resistant traits through epigenetics or maternal effects (Gauci et al. 2024; Martins et al. 2019), which may lead to the development of cellular mechanisms that permit them to respond better to stress. For instance, enhanced heat shock protein expression (Jueterbock et al. 2014; Smolina et al. 2016) and an increased capacity to change membrane fluidity (Britton et al. 2020; Los and Murata 2004) are potential mechanisms by which individuals may cope with thermal stress. Enhanced thermal tolerance also has been observed for individuals hosting putatively beneficial microbial communities (Allsup et al. 2023; Ziegler et al. 2017). In *Phyllospora*, some bacteria have been associated with heat‐resistant seaweed host *loci* (Wood et al. 2022), but experimental manipulations of these microorganisms are needed to determine if they indeed affect host responses to stress (Marzinelli et al. 2024; McGrath et al. 2024) and heat tolerance (e.g., Jongen et al. 2025). Identifying the mechanisms linked to extreme‐heat tolerance of survivors could be key to conserving habitat‐forming seaweeds in the future, for instance, via developing assisted gene flow strategies (Wood et al. 2024) if mechanisms are linked to genetic characteristics.

While extreme MHWs had adverse effects on *Phyllospora* early life‐stages, we observed no measurable negative (or positive) effects on either germlings or juveniles when exposed to the strong MHW. This response is supported by field surveys from the warm‐edge population, which showed no significant declines in natural populations in response to moderate and strong MHWs (Davis et al., in review). A maximum of 23°C represents a strong winter MHW, but this is still within the range of fucoid thermal tolerance (Lüning 1984) and close to the optimal temperature for *Phyllospora* photosynthesis (i.e., 22.3°C Veenhof et al. 2024), which might explain similar responses under the strong MHW and ambient conditions. In this context, the strong MHW did not clearly impact pigment and C:N ratios of the juveniles, which is consistent with the observed lack of patterns in condition and photosynthesis. These results contrast with previous studies, which have revealed higher C:N ratios (Lowman et al. 2022) and lower pigment content (Kumar et al. 2020; Liu et al. 2017) of seaweeds subjected to higher temperatures (but see Castro et al. 2024). Still, we observed high C:N ratios in the juveniles under a MHW and ambient conditions, indicating a possible nitrogen limitation, reduction in nitrogen uptake, or increased stored nitrogen assimilation due to stress (Hurd et al. 2014; Sheng et al. 2025; Smart et al. 2022).

This study emphasises the high vulnerability of *Phyllospora* to extreme MHWs, especially closer to the warm‐edge, where temperatures are closer to these extreme scenarios (24°C–25°C in summer). Individuals that survive in an extreme MHW scenario (10%–30%) might still not be present in all populations or be sufficient to sustain the population dynamics, risking the loss of genetic diversity in the phase of extreme events, especially in populations under a higher threat of environmental change and concurrent decline (Young et al. 2023). Nevertheless, under strong MHWs, significant mortalities are not expected, offering a window of opportunity to conserve populations at risk while extreme events become more common (Oliver et al. 2019). Further research towards this conservation goal, with interventions such as assisted evolution (Wood et al. 2024) or biobanking (to store genetic diversity in the long term, Day and Stacey 2008) is necessary to enhance resilience in the face of extreme events (i.e., future‐proofing, Coleman et al. 2020; Wood et al. 2019).

## Author Contributions

**Catalina A. Musrri:** conceptualization (equal), data curation (lead), formal analysis (lead), investigation (lead), methodology (equal), resources (equal), validation (equal), visualization (lead), writing – original draft (lead), writing – review and editing (equal). **Georgina Wood:** conceptualization (equal), investigation (equal), methodology (equal), resources (equal), supervision (equal), validation (supporting), visualization (supporting), writing – original draft (supporting), writing – review and editing (equal). **Adriana Vergés:** conceptualization (equal), funding acquisition (equal), investigation (supporting), methodology (equal), project administration (equal), resources (equal), supervision (equal), validation (equal), visualization (supporting), writing – original draft (supporting), writing – review and editing (equal). **Damon Britton:** conceptualization (equal), investigation (supporting), methodology (equal), writing – review and editing (equal). **Catriona L. Hurd:** conceptualization (equal), investigation (supporting), methodology (equal), writing – review and editing (equal). **Ezequiel M. Marzinelli:** conceptualization (equal), formal analysis (supporting), funding acquisition (equal), investigation (supporting), methodology (equal), project administration (equal), resources (equal), supervision (equal), validation (equal), visualization (supporting), writing – original draft (supporting), writing – review and editing (equal).

## Funding

This research was supported by the Lim Sutton Initiative, the Ian Potter Foundation and an Australian Research Council grant to EMM, AV and GW (LP240200762). CAM was supported by an Australian Government International Research Training Program (RTP) scholarship and the Marine Studies Institute (MSI) Ruhm Award in Marine Ecology. GW was supported by an Australian Research Council (ARC) ARC Industry Fellowship (IE230100464). The University of Sydney facilitated open access publishing.

## Disclosure

Statement on inclusion: Our study brings together authors from different countries and backgrounds, all based in the country where this study was developed. This work was developed in the land and waters of the Dharawal, Gadigal, Darkinjung, Worimi and Bidjigal People. We pay our respects to their people and cultures, past and present. While this specific work did not involve direct participation of Aboriginal people, the overarching project ‘Operation Crayweed’ does involve a close partnership with the Gamay Rangers First Nations group.

## Conflicts of Interest

The authors declare no conflicts of interest.

## Supporting information


**Data S1:** ece372998‐sup‐0001‐supinfo.docx.

## Data Availability

This work data and code are available in the Dryad Digital Repository https://doi.org/10.5061/dryad.7pvmcvf62.

## References

[ece372998-bib-0001] Al‐Janabi, B. , I. Kruse , A. Graiff , U. Karsten , and M. Wahl . 2016. “Genotypic Variation Influences Tolerance to Warming and Acidification of Early Life‐Stage *Fucus vesiculosus* L. (Phaeophyceae) in a Seasonally Fluctuating Environment.” Marine Biology 163: 14. 10.1007/s00227-015-2804-8.

[ece372998-bib-0002] Allsup, C. M. , I. George , and R. A. Lankau . 2023. “Shifting Microbial Communities Can Enhance Tree Tolerance to Changing Climates.” Science 380: 835–840. 10.1126/science.adf2027. 37228219

[ece372998-bib-0003] Alsuwaiyan, N. A. , S. Vranken , K. Filbee‐Dexter , M. Cambridge , M. A. Coleman , and T. Wernberg . 2021. “Genotypic Variation in Response to Extreme Events May Facilitate Kelp Adaptation Under Future Climates.” Marine Ecology Progress Series 672: 111–121. 10.3354/meps13802.

[ece372998-bib-0004] Andrew, N. 1999. Under Southern Seas: The Ecology of Australia's Rocky Reefs. UNSW Press.

[ece372998-bib-0005] Andrews, S. , S. Bennett , and T. Wernberg . 2014. “Reproductive Seasonality and Early Life Temperature Sensitivity Reflect Vulnerability of a Seaweed Undergoing Range Reduction.” Marine Ecology Progress Series 495: 119–129. 10.3354/meps10567.

[ece372998-bib-0006] Bahr, K. D. , K. S. Rodgers , and P. L. Jokiel . 2018. “Ocean Warming Drives Decline in Coral Metabolism While Acidification Highlights Species‐Specific Responses.” Marine Biology Research 14: 924–935. 10.1080/17451000.2018.1551616.

[ece372998-bib-0008] Bennett, S. , R. Vaquer‐Sunyer , G. Jordá , M. Forteza , G. Roca , and N. Marbà . 2022. “Thermal Performance of Seaweeds and Seagrasses Across a Regional Climate Gradient.” Frontiers in Marine Science 9: 733315. 10.3389/fmars.2022.733315.

[ece372998-bib-0009] Bennett, S. , T. Wernberg , B. Arackal Joy , T. de Bettignies , and A. H. Campbell . 2015. “Central and Rear‐Edge Populations Can Be Equally Vulnerable to Warming.” Nature Communications 6: 10280. 10.1038/ncomms10280. PMC4703895 26691184

[ece372998-bib-0010] Bernardello, R. , E. Serrano , R. Coma , M. Ribes , and N. Bahamon . 2016. “A Comparison of Remote‐Sensing SST and In Situ Seawater Temperature in Near‐Shore Habitats in the Western Mediterranean Sea.” Marine Ecology Progress Series 559: 21–34. 10.3354/meps11896.

[ece372998-bib-0011] Britton, D. , M. Schmid , F. Noisette , et al. 2020. “Adjustments in Fatty Acid Composition Is a Mechanism That Can Explain Resilience to Marine Heatwaves and Future Ocean Conditions in the Habitat‐Forming Seaweed *Phyllospora Comosa* (Labillardière) C.Agardh.” Global Change Biology 26: 3512–3524. 10.1111/gcb.15052. 32105368

[ece372998-bib-0012] Capdevila, P. , B. Hereu , R. Salguero‐Gómez , et al. 2019. “Warming Impacts on Early Life Stages Increase the Vulnerability and Delay the Population Recovery of a Long‐Lived Habitat‐Forming Macroalga.” Journal of Ecology 107: 1129–1140. 10.1111/1365-2745.13090.

[ece372998-bib-0013] Castro, L. C. , A. Vergés , S. C. Straub , et al. 2024. “Effect of Marine Heatwaves and Warming on Kelp Microbiota Influence Trophic Interactions.” Molecular Ecology 33: e17267. 10.1111/mec.17267. 38230446

[ece372998-bib-0014] Clark, J. S. , A. G. B. Poore , M. A. Coleman , and M. A. Doblin . 2020. “Local Scale Thermal Environment and Limited Gene Flow Indicates Vulnerability of Warm Edge Populations in a Habitat Forming Macroalga.” Frontiers in Marine Science 7: 711. 10.3389/fmars.2020.00711.

[ece372998-bib-0015] Clark, J. S. , A. G. B. Poore , and M. A. Doblin . 2018. “Shaping Up for Stress: Physiological Flexibility Is Key to Survivorship in a Habitat‐Forming Macroalga.” Journal of Plant Physiology 231: 346–355. 10.1016/j.jplph.2018.10.005. 30388674

[ece372998-bib-0016] Coleman, M. A. , and T. Wernberg . 2017. “Forgotten Underwater Forests: The Key Role of Fucoids on Australian Temperate Reefs.” Ecology and Evolution 7: 8406–8418. 10.1002/ece3.3279. 29075458 PMC5648665

[ece372998-bib-0017] Coleman, M. A. , G. Wood , K. Filbee‐Dexter , et al. 2020. “Restore or Redefine: Future Trajectories for Restoration.” Frontiers in Marine Science 7: 237. 10.3389/fmars.2020.00237.

[ece372998-bib-0018] Cumming, E. E. , T. G. Matthews , C. J. Sanderson , B. A. Ingram , and A. Bellgrove . 2019. “Optimal Spawning Conditions of *Phyllospora Comosa* (Phaeophyceae, Fucales) for Mariculture.” Journal of Applied Phycology 31: 3041–3050. 10.1007/s10811-019-01788-8.

[ece372998-bib-0019] Day, J. G. , and G. N. Stacey . 2008. “Biobanking.” Molecular Biotechnology 40: 202–213. 10.1007/s12033-008-9099-7. 18780190

[ece372998-bib-0020] Doherty, M. L. , J. V. Johnson , and G. Goodbody‐Gringley . 2025. “Widespread Coral Bleaching and Mass Mortality During the 2023–2024 Marine Heatwave in Little Cayman.” PLoS One 20: e0322636. 10.1371/journal.pone.0322636. 40315251 PMC12047782

[ece372998-bib-0021] Doney, S. C. , M. Ruckelshaus , J. Emmett Duffy , et al. 2012. “Climate Change Impacts on Marine Ecosystems.” Annual Review of Marine Science 4: 11–37. 10.1146/annurev-marine-041911-111611. 22457967

[ece372998-bib-0022] Edwards, M. S. 2022. “It's the Little Things: The Role of Microscopic Life Stages in Maintaining Kelp Populations.” Frontiers in Marine Science 9: 871204. 10.3389/fmars.2022.871204.

[ece372998-bib-0023] Eger, A. M. , E. M. Marzinelli , R. Beas‐Luna , et al. 2023. “The Value of Ecosystem Services in Global Marine Kelp Forests.” Nature Communications 14: 1894. 10.1038/s41467-023-37385-0. PMC10113392 37072389

[ece372998-bib-0024] Eggert, A. 2012. “Seaweed Responses to Temperature.” In Seaweed Biology: Novel Insights Into Ecophysiology, Ecology and Utilization, Ecological Studies, edited by C. Wiencke and K. Bischof , 47–66. Springer. 10.1007/978-3-642-28451-9_3.

[ece372998-bib-0025] Ferreira, J. G. , F. Arenas , B. Martínez , S. J. Hawkins , and S. R. Jenkins . 2014. “Physiological Response of Fucoid Algae to Environmental Stress: Comparing Range Centre and Southern Populations.” New Phytologist 202: 1157–1172. 10.1111/nph.12749. 24580117

[ece372998-bib-0026] Filbee‐Dexter, K. , T. Wernberg , S. P. Grace , et al. 2020. “Marine Heatwaves and the Collapse of Marginal North Atlantic Kelp Forests.” Scientific Reports 10: 13388. 10.1038/s41598-020-70273-x. 32770015 PMC7414212

[ece372998-bib-0027] Flukes, E. B. , J. T. Wright , and C. R. Johnson . 2015. “Phenotypic Plasticity and Biogeographic Variation in Physiology of Habitat‐Forming Seaweed: Response to Temperature and Nitrate.” Journal of Phycology 51: 896–909. 10.1111/jpy.12330. 26986886

[ece372998-bib-0028] Fox, J. , S. Weisberg , and B. Price . 2001. “Car: Companion to Applied Regression.” 10.32614/CRAN.package.car.

[ece372998-bib-0029] Fredersdorf, J. , R. Müller , S. Becker , C. Wiencke , and K. Bischof . 2009. “Interactive Effects of Radiation, Temperature and Salinity on Different Life History Stages of the Arctic Kelp *Alaria esculenta* (Phaeophyceae).” Oecologia 160: 483–492. 10.1007/s00442-009-1326-9. 19330357

[ece372998-bib-0030] Gauci, C. , A. Jueterbock , A. Khatei , G. Hoarau , and I. Bartsch . 2024. “Thermal Priming of *Saccharina Latissima*: A Promising Strategy to Improve Seaweed Production and Restoration in Future Climates.” Marine Ecology Progress Series 745: 59–71. 10.3354/meps14683.

[ece372998-bib-0031] Harley, C. D. G. 2011. “Climate Change, Keystone Predation, and Biodiversity Loss.” Science 334: 1124–1127. 10.1126/science.1210199. 22116885

[ece372998-bib-0032] Harris, R. 2024. Bridging Land and Sea Perspectives of Photosynthetic Thermal Tolerance in a Climate Change Context (Doctor of Philosophy). Division of Ecology and Evolution Research School of Biology, Australian National University.

[ece372998-bib-0033] He, Q. , and B. R. Silliman . 2019. “Climate Change, Human Impacts, and Coastal Ecosystems in the Anthropocene.” Current Biology 29: R1021–R1035. 10.1016/j.cub.2019.08.042. 31593661

[ece372998-bib-0034] Hernández, S. , A. G. García , F. Arenas , et al. 2023. “Range‐Edge Populations of Seaweeds Show Niche Unfilling and Poor Adaptation to Increased Temperatures.” Journal of Biogeography 50, no. 4: 780–791. 10.1111/jbi.14572.

[ece372998-bib-0035] Hobday, A. J. , L. V. Alexander , S. E. Perkins , et al. 2016. “A Hierarchical Approach to Defining Marine Heatwaves.” Progress in Oceanography 141: 227–238. 10.1016/j.pocean.2015.12.014.

[ece372998-bib-0036] Hobday, A. J. , E. C. J. Oliver , A. S. Gupta , et al. 2018. “Categorizing and Naming Marine Heatwaves.” Oceanography 31: 162–173.

[ece372998-bib-0037] Hoey, A. S. , E. Howells , J. L. Johansen , et al. 2016. “Recent Advances in Understanding the Effects of Climate Change on Coral Reefs.” Diversity 8: 12. 10.3390/d8020012.

[ece372998-bib-0038] Hurd, C. L. , P. J. Harrison , K. Bischof , and C. S. Lobban . 2014. Seaweed Ecology and Physiology. 2nd ed. Cambridge University Press. 10.1017/CBO9781139192637.

[ece372998-bib-0039] Jongen, R. , E. M. Marzinelli , S. Vadillo Gonzalez , J. L. Hart , S. Kjelleberg , and P. E. Gribben . 2025. “Contrasting Effects of Rhizosphere and Sediment Microbiota on Seagrass Performance in Response to a Simulated Marine Heatwave.” Journal of Ecology 113: 1–18. 10.1111/1365-2745.70104.

[ece372998-bib-0040] Jueterbock, A. , S. Kollias , I. Smolina , et al. 2014. “Thermal Stress Resistance of the Brown Alga *Fucus serratus* Along the North‐Atlantic Coast: Acclimatization Potential to Climate Change.” Marine Genomics 13: 27–36. 10.1016/j.margen.2013.12.008. 24393606

[ece372998-bib-0041] King, N. G. , T. Leathers , K. E. Smith , and D. A. Smale . 2025. “The Influence of Pre‐Exposure to Marine Heatwaves on the Critical Thermal Maxima (CTmax) of Marine Foundation Species.” Functional Ecology 39: 1869–1878. 10.1111/1365-2435.14622.

[ece372998-bib-0042] King, N. G. , N. J. McKeown , D. A. Smale , et al. 2019. “Evidence for Different Thermal Ecotypes in Range Centre and Trailing Edge Kelp Populations.” Journal of Experimental Marine Biology and Ecology 514–515: 10–17. 10.1016/j.jembe.2019.03.004.

[ece372998-bib-0043] Kumar, Y. N. , S.‐W. Poong , C. Gachon , J. Brodie , A. Sade , and P.‐E. Lim . 2020. “Impact of Elevated Temperature on the Physiological and Biochemical Responses of *Kappaphycus Alvarezii* (Rhodophyta).” PLoS One 15: e0239097. 10.1371/journal.pone.0239097. 32925956 PMC7489555

[ece372998-bib-0044] Laeseke, P. , B. D.‐C. Martínez , and K. Bischof . 2024. “Large‐Scale Deviations Between Realized and Fundamental Thermal Niches in Global Seaweed Distributions.” Diversity and Distributions 30, no. 8: e13868. 10.1111/ddi.13868.

[ece372998-bib-0045] Lenth, R. V. 2017. “Emmeans: Estimated Marginal Means, Aka Least‐Squares Means.” 10.32614/CRAN.package.emmeans.

[ece372998-bib-0046] Liu, C. , D. Zou , Y. Yang , B. Chen , and H. Jiang . 2017. “Temperature Responses of Pigment Contents, Chlorophyll Fluorescence Characteristics, and Antioxidant Defenses in *Gracilariopsis Lemaneiformis* (Gracilariales, Rhodophyta) Under Different CO2 Levels.” Journal of Applied Phycology 29: 983–991. 10.1007/s10811-016-0971-8.

[ece372998-bib-0047] Lloret, F. , and E. Batllori . 2021. “Climate‐Induced Global Forest Shifts due to Heatwave‐Drought.” In Ecosystem Collapse and Climate Change, edited by J. G. Canadell and R. B. Jackson , 155–186. Springer International Publishing. 10.1007/978-3-030-71330-0_7.

[ece372998-bib-0048] Logan, B. A. , W. W. Adams , and B. Demmig‐Adams . 2007. “Avoiding Common Pitfalls of Chlorophyll Fluorescence Analysis Under Field Conditions.” Functional Plant Biology 34, no. 9: 853–859. 10.1071/FP07113. 32689413

[ece372998-bib-0049] López‐Goldar, X. , and A. A. Agrawal . 2021. “Ecological Interactions, Environmental Gradients, and Gene Flow in Local Adaptation.” Trends in Plant Science 26: 796–809. 10.1016/j.tplants.2021.03.006. 33865704

[ece372998-bib-0050] Los, D. A. , and N. Murata . 2004. “Membrane Fluidity and Its Roles in the Perception of Environmental Signals. Biochim. Biophys. Acta—Biomembr.” Lipid‐Protein Interactions 1666: 142–157. 10.1016/j.bbamem.2004.08.002. 15519313

[ece372998-bib-0051] Lotze, H. K. , B. Worm , and U. Sommer . 2001. “Strong Bottom‐Up and Top‐Down Control of Early Life Stages of Macroalgae.” Limnology and Oceanography 46: 749–757. 10.4319/lo.2001.46.4.0749.

[ece372998-bib-0052] Lowman, H. E. , K. A. Emery , J. E. Dugan , and R. J. Miller . 2022. “Nutritional Quality of Giant Kelp Declines due to Warming Ocean Temperatures.” Oikos 2022: 1–14. 10.1111/oik.08619.

[ece372998-bib-0053] Lüning, K. 1984. “Temperature Tolerance and Biogeography of Seaweeds: The Marine Algal Flora of Helgoland (North Sea) as an Example.” Helgoländer Wissenschaftliche Meeresuntersuchungen 38: 305–317. 10.1007/BF01997486.

[ece372998-bib-0054] Margalef‐Marrase, J. , M. Á. Pérez‐Navarro , and F. Lloret . 2020. “Relationship Between Heatwave‐Induced Forest Die‐Off and Climatic Suitability in Multiple Tree Species.” Global Change Biology 26: 3134–3146. 10.1111/gcb.15042. 32064733

[ece372998-bib-0055] Martínez, B. , F. Arenas , M. Rubal , et al. 2012. “Physical Factors Driving Intertidal Macroalgae Distribution: Physiological Stress of a Dominant Fucoid at Its Southern Limit.” Oecologia 170: 341–353. 10.1007/s00442-012-2324-x. 22526940

[ece372998-bib-0056] Martínez, B. , B. Radford , M. S. Thomsen , et al. 2018. “Distribution Models Predict Large Contractions of Habitat‐Forming Seaweeds in Response to Ocean Warming.” Diversity and Distributions 24: 1350–1366. 10.1111/ddi.12767.

[ece372998-bib-0057] Martins, N. , G. A. Pearson , and L. Gouveia . 2019. “Hybrid Vigour for Thermal Tolerance in Hybrids Between the Allopatric Kelps *Laminaria Digitata* and *L. pallida* (Laminariales, Phaeophyceae) With Contrasting Thermal Affinities.” European Journal of Phycology 54: 548–561. 10.1080/09670262.2019.1613571.

[ece372998-bib-0058] Marzinelli, E. M. , A. H. Campbell , A. Vergés , M. A. Coleman , B. P. Kelaher , and P. D. Steinberg . 2014. “Restoring Seaweeds: Does the Declining Fucoid *Phyllospora Comosa* Support Different Biodiversity Than Other Habitats?” Journal of Applied Phycology 26: 1089–1096. 10.1007/s10811-013-0158-5.

[ece372998-bib-0059] Marzinelli, E. M. , M. R. Leong , A. H. Campbell , P. D. Steinberg , and A. Vergés . 2016. “Does Restoration of a Habitat‐Forming Seaweed Restore Associated Faunal Diversity?” Restoration Ecology 24: 81–90. 10.1111/rec.12292.

[ece372998-bib-0060] Marzinelli, E. M. , T. Thomas , S. Vadillo Gonzalez , S. Egan , and P. D. Steinberg . 2024. “Seaweeds as Holobionts: Current State, Challenges, and Potential Applications.” Journal of Phycology 60: 785–796. 10.1111/jpy.13485. 39047050

[ece372998-bib-0061] McCarthy, O. S. , M. W. Pomeroy , and J. E. Smith . 2024. “Corals That Survive Repeated Thermal Stress Show Signs of Selection and Acclimatization.” PLoS One 19: e0303779. 10.1371/journal.pone.0303779. 39083457 PMC11290665

[ece372998-bib-0062] McGrath, A. H. , K. Lema , S. Egan , et al. 2024. “Disentangling Direct vs Indirect Effects of Microbiome Manipulations in a Habitat‐Forming Marine Holobiont.” NPJ Biofilms and Microbiomes 10: 33. 10.1038/s41522-024-00503-x. 38553475 PMC10980776

[ece372998-bib-0063] Mota, C. F. , A. H. Engelen , E. A. Serrao , et al. 2018. “Differentiation in Fitness‐Related Traits in Response to Elevated Temperatures Between Leading and Trailing Edge Populations of Marine Macrophytes.” PLoS One 13: e0203666. 10.1371/journal.pone.0203666. 30212558 PMC6136734

[ece372998-bib-0064] Nielsen, S. L. , H. D. Nielsen , and M. F. Pedersen . 2014. “Juvenile Life Stages of the Brown Alga *Fucus serratus* L. Are More Sensitive to Combined Stress From High Copper Concentration and Temperature Than Adults.” Marine Biology 161: 1895–1904. 10.1007/s00227-014-2471-1.

[ece372998-bib-0065] Oliver, E. C. J. , M. T. Burrows , M. G. Donat , et al. 2019. “Projected Marine Heatwaves in the 21st Century and the Potential for Ecological Impact.” Frontiers in Marine Science 6: 734. 10.3389/fmars.2019.00734.

[ece372998-bib-0066] Pearson, G. A. , A. Lago‐Leston , and C. Mota . 2009. “Frayed at the Edges: Selective Pressure and Adaptive Response to Abiotic Stressors Are Mismatched in Low Diversity Edge Populations.” Journal of Ecology 97: 450–462. 10.1111/j.1365-2745.2009.01481.x.

[ece372998-bib-0067] R Core Team . 2024. R: A Language and Environment for Statistical Computing. R Foundation for Statistical Computing. https://www.R‐project.org/.

[ece372998-bib-0068] Schiel, D. R. , and M. S. Foster . 2006. “The Population Biology of Large Brown Seaweeds: Ecological Consequences of Multiphase Life Histories in Dynamic Coastal Environments.” Annual Review of Ecology, Evolution, and Systematics 37: 343–372. 10.1146/annurev.ecolsys.37.091305.110251.

[ece372998-bib-0069] Schneider, C. A. , W. S. Rasband , and K. W. Eliceiri . 2012. “NIH Image to ImageJ: 25 Years of Image Analysis.” Nature Methods 9: 671–675. 10.1038/nmeth.2089. 22930834 PMC5554542

[ece372998-bib-0070] Schwoerbel, J. , W. Visch , J. T. Wright , A. Bellgrove , J. C. Sanderson , and C. L. Hurd . 2024. “Thermal Performance Curves Identify Seasonal and Site‐Specific Variation in the Development of *Ecklonia radiata* (Phaeophyceae) Gametophytes and Sporophytes.” Journal of Phycology 60: 83–101. 10.1111/jpy.13406. 37897074

[ece372998-bib-0071] Seely, G. R. , M. J. Duncan , and W. E. Vidaver . 1972. “Preparative and Analytical Extraction of Pigments From Brown Algae With Dimethyl Sulfoxide.” Marine Biology 12: 184–188. 10.1007/BF00350754.

[ece372998-bib-0072] Sheng, X. , X. Zuo , L. Luo , et al. 2025. “Impact of Carbon and Nitrogen Assimilation in *Sargassum Fusiforme* (Harvey) Setchell due to Marine Heatwave Under Global Warming.” Global Change Biology 31: e70074. 10.1111/gcb.70074. 39981658

[ece372998-bib-0073] Smale, D. A. 2020. “Impacts of Ocean Warming on Kelp Forest Ecosystems.” New Phytologist 225: 1447–1454. 10.1111/nph.16107. 31400287

[ece372998-bib-0074] Smale, D. A. , M. T. Burrows , P. Moore , N. O'Connor , and S. J. Hawkins . 2013. “Threats and Knowledge Gaps for Ecosystem Services Provided by Kelp Forests: A Northeast Atlantic Perspective.” Ecology and Evolution 3: 4016–4038. 10.1002/ece3.774. 24198956 PMC3810891

[ece372998-bib-0075] Smale, D. A. , and T. Wernberg . 2009. “Satellite‐Derived SST Data as a Proxy for Water Temperature in Nearshore Benthic Ecology.” Marine Ecology Progress Series 387: 27–37. 10.3354/meps08132.

[ece372998-bib-0076] Smale, D. A. , and T. Wernberg . 2013. “Extreme Climatic Event Drives Range Contraction of a Habitat‐Forming Species.” Proceedings of the Royal Society B: Biological Sciences 280: 20122829. 10.1098/rspb.2012.2829. PMC3574333 23325774

[ece372998-bib-0077] Smart, J. N. , M. Schmid , E. R. Paine , D. Britton , A. Revill , and C. L. Hurd . 2022. “Seasonal Ammonium Uptake Kinetics of Four Brown Macroalgae: Implications for Use in Integrated Multi‐Trophic Aquaculture.” Journal of Applied Phycology 34: 1693–1708. 10.1007/s10811-022-02743-w.

[ece372998-bib-0078] Smith, K. E. , M. T. Burrows , A. J. Hobday , et al. 2023. “Biological Impacts of Marine Heatwaves.” Annual Review of Marine Science 15: 119–145. 10.1146/annurev-marine-032122-121437. 35977411

[ece372998-bib-0079] Smolina, I. , S. Kollias , A. Jueterbock , J. A. Coyer , and G. Hoarau . 2016. “Variation in Thermal Stress Response in Two Populations of the Brown Seaweed, *Fucus distichus* , From the Arctic and Subarctic Intertidal.” Royal Society Open Science 3: 150429. 10.1098/rsos.150429. 26909170 PMC4736925

[ece372998-bib-0080] Starko, S. , M. van der Mheen , A. Pessarrodona , et al. 2024. “Impacts of Marine Heatwaves in Coastal Ecosystems Depend on Local Environmental Conditions.” Global Change Biology 30: e17469. 10.1111/gcb.17469. 39155748

[ece372998-bib-0081] Steneck, R. S. , M. H. Graham , B. J. Bourque , et al. 2002. “Kelp Forest Ecosystems: Biodiversity, Stability, Resilience and Future.” Environmental Conservation 29: 436–459. 10.1017/S0376892902000322.

[ece372998-bib-0082] Stobart, B. , S. Mayfield , C. Mundy , A. J. Hobday , and J. R. Hartog . 2015. “Comparison of In Situ and Satellite Sea Surface‐Temperature Data From South Australia and Tasmania: How Reliable Are Satellite Data as a Proxy for Coastal Temperatures in Temperate Southern Australia?” Marine and Freshwater Research 67: 612–625. 10.1071/MF14340.

[ece372998-bib-0083] Straub, S. C. , T. Wernberg , E. M. Marzinelli , A. Vergés , B. P. Kelaher , and M. A. Coleman . 2022. “Persistence of Seaweed Forests in the Anthropocene Will Depend on Warming and Marine Heatwave Profiles.” Journal of Phycology 58: 22–35. 10.1111/jpy.13222. 34800039

[ece372998-bib-0084] Teagle, H. , S. J. Hawkins , P. J. Moore , and D. A. Smale . 2017. “The Role of Kelp Species as Biogenic Habitat Formers in Coastal Marine Ecosystems.” Journal of Experimental Marine Biology and Ecology 492: 81–98. 10.1016/j.jembe.2017.01.017.

[ece372998-bib-0085] Thomsen, M. S. , L. Mondardini , T. Alestra , et al. 2019. “Local Extinction of Bull Kelp (*Durvillaea* spp.) due to a Marine Heatwave.” Frontiers in Marine Science 6: 84. 10.3389/fmars.2019.00084.

[ece372998-bib-0086] Underwood, A. J. , M. J. Kingsford , and N. L. Andrew . 1991. “Patterns in Shallow Subtidal Marine Assemblages Along the Coast of New South Wales.” Australian Journal of Ecology 16: 231–249. 10.1111/j.1442-9993.1991.tb01050.x.

[ece372998-bib-0087] Veenhof, R. J. , M. A. Coleman , C. Champion , et al. 2024. “Novel High‐Throughput Oxygen Saturation Measurements for Quantifying the Physiological Performance of Macroalgal Early Life Stages.” Journal of Phycology 60: 1161–1172. 10.1111/jpy.13489. 39105657

[ece372998-bib-0088] Wernberg, T. , S. Bennett , R. C. Babcock , et al. 2016. “Climate‐Driven Regime Shift of a Temperate Marine Ecosystem.” Science 353: 169–172. 10.1126/science.aad8745. 27387951

[ece372998-bib-0089] Wernberg, T. , T. de Bettignies , B. A. Joy , and P. M. Finnegan . 2016. “Physiological Responses of Habitat‐Forming Seaweeds to Increasing Temperatures.” Limnology and Oceanography 62: 2180–2190. 10.1002/lno.10362.

[ece372998-bib-0090] Wernberg, T. , K. Krumhansl , K. Filbee‐Dexter , and M. F. Pedersen . 2019. “Chapter 3—Status and Trends for the World's Kelp Forests.” In World Seas: An Environmental Evaluation, edited by C. Sheppard , 2nd ed., 57–78. Academic Press. 10.1016/B978-0-12-805052-1.00003-6.

[ece372998-bib-0091] Wood, G. , E. M. Marzinelli , A. H. Campbell , P. D. Steinberg , A. Vergés , and M. A. Coleman . 2021. “Genomic Vulnerability of a Dominant Seaweed Points to Future‐Proofing Pathways for Australia's Underwater Forests.” Global Change Biology 27: 2200–2212. 10.1111/gcb.15534. 33511779

[ece372998-bib-0092] Wood, G. , E. M. Marzinelli , M. A. Coleman , et al. 2019. “Restoring Subtidal Marine Macrophytes in the Anthropocene: Trajectories and Future‐Proofing.” Marine and Freshwater Research 70: 936–951. 10.1071/MF18226.

[ece372998-bib-0093] Wood, G. , P. D. Steinberg , A. H. Campbell , A. Vergés , M. A. Coleman , and E. M. Marzinelli . 2022. “Host Genetics, Phenotype and Geography Structure the Microbiome of a Foundational Seaweed.” Molecular Ecology 31: 2189–2206. 10.1111/mec.16378. 35104026 PMC9540321

[ece372998-bib-0094] Wood, G. V. , K. J. Griffin , M. van der Mheen , et al. 2024. “Reef Adapt: A Tool to Inform Climate‐Smart Marine Restoration and Management Decisions.” Communications Biology 7: 1–12. 10.1038/s42003-024-06970-4. 39478133 PMC11526119

[ece372998-bib-0095] Young, M. A. , K. Critchell , A. D. Miller , et al. 2023. “Mapping the Impacts of Multiple Stressors on the Decline in Kelps Along the Coast of Victoria, Australia.” Diversity and Distributions 29: 199–220. 10.1111/ddi.13654.

[ece372998-bib-0096] Ziegler, M. , F. O. Seneca , L. K. Yum , S. R. Palumbi , and C. R. Voolstra . 2017. “Bacterial Community Dynamics Are Linked to Patterns of Coral Heat Tolerance.” Nature Communications 8: 14213. 10.1038/ncomms14213. PMC5309854 28186132

[ece372998-bib-0097] Zuur, A. F. , E. N. Ieno , and C. S. Elphick . 2010. “A Protocol for Data Exploration to Avoid Common Statistical Problems.” Methods in Ecology and Evolution 1: 3–14. 10.1111/j.2041-210X.2009.00001.x.

