# Supplementary material for: Response of Early Life‐Stages of Forest‐Forming Seaweeds From Warm‐Edge and Central Populations to Marine Heatwaves
Source: Ecol Evol. 2026 Jan 29;16(2):e72998. doi: 10.1002/ece3.72998 (PMC12854809; doi:10.1002/ece3.72998)
Supplement: Supplementary file 1 — Data S1: ece372998‐sup‐0001‐supinfo.docx. [file ECE3-16-e72998-s001.docx]

**Supplementary Information**

**Ecology and Evolution** - Response of early life-stages of forest-forming seaweeds from warm-edge and central populations to marine heatwaves

Catalina A. Musrri^1*^, Georgina Wood^2,3^, Adriana Vergés^4^, Damon Britton^5^, Catriona L. Hurd^5^, Ezequiel M. Marzinelli^1^

^1^ The University of Sydney, School of Life and Environmental Sciences, Sydney, NSW 2006, Australia

^2^ College of Science and Engineering, Flinders University, Adelaide SA, Australia 5001

^3^ UWA Oceans Institute and School of Biological Sciences, University of Western Australia, Perth, WA, Australia

^4^ Centre for Marine Science and Innovation, School of Biological, Earth and Environmental Sciences, UNSW Sydney, Sydney, NSW, Australia

^5^ Institute for Marine and Antarctic Studies (IMAS), University of Tasmania (UTAS), Battery Point, Tasmania, Australia

*Corresponding author: Catalina A. Musrri

Email: [catalina.musrrifuenzalida@sydney.edu.au](mailto:catalina.musrrifuenzalida@sydney.edu.au)

Phone: +61415686155

Address: Level 6, F22 Life, Earth and Environmental Sciences (LEES) Building, City Rd &, Eastern Ave, Camperdown NSW 2050

| **Page** | **Supplementary Figure/Table** |
| --- | --- |
| 4 | Figure S1: Visual differences between (a) female and (b) male *Phyllospora comosa* blades. |
| 5 | Figure S2: Summary of Phyllospora comosa germling experiment. |
| 6 | Figure S3: Phyllospora comosa germling measurements to record a) length (mm) and b) area (mm^2^). |
| 6 | Figure S4: Phyllospora comosa germlings shape categories a) elongated, b) branched, c) rounded. |
| 7 | Figure S5: Phyllospora comosa juvenile full individual length example. |
| 8 | Figure S6: Summary of *Phyllospora comosa* juvenile experiment |
| 9 | Figure S7: Example of condition metrics recorded in *Phyllospora comosa* juveniles. |
| 10 | Figure S8: Proportion of *Phyllospora comosa* germlings with different shapes (n = 6). |
| 11 | Figure S*9*: Relative growth rate (RGR %d^-1^; mean +\- SE; n = 8-12) in *Phyllospora comosa* juveniles from four populations along New South Wales |
| 12 | Figure S*10*: Phyllospora comosa juvenile condition metrics: bleaching, fouling, necrosis and green colouration (mean, +\- SE, n = 6) under experimental MHW scenarios. |
| 13 | Table S1: Summary of strong and severe MHW events in four sites along New South Wales. |
| 14 | Table S2: Summary of *Phyllospora comosa* germling experiment results. |
| 15 | Table S3: Differences in *Phyllospora comosa* germling survival (%) from different populations (Black Head and Palm Beach) and under three MHW scenarios (Ambient, strong and extreme MHW) after 9, 16, 23 and 30 days. |
| 16 | Table S4: Differences in *Phyllospora comosa* germling area (mm^2^) from different populations (Black Head and Palm Beach) and under three MHW scenarios (Ambient, strong and extreme MHW) after 9, 16, 23 and 30 days. |
| 17 | Table S5: Differences in *Phyllospora comosa* germling length (mm) from different populations (Black Head and Palm Beach) and under three MHW scenarios (Ambient, strong and extreme MHW) after 9, 16, 23 and 30 days. |
| 18 | Table S6: Differences in *Phyllospora comosa* germlings with elongated shape (%) from different populations (Black Head and Palm Beach) and under three MHW scenarios (Ambient, strong and extreme MHW) after 9, 16, 23 and 30 days |
| 19 | Table S7: Differences in *Phyllospora comosa* germlings with branched shape (%) from different populations (Black Head and Palm Beach) and under three MHW scenarios (Ambient, strong and extreme MHW) after 9, 16, 23 and 30 days. |
| 20 | Table S8: Differences in *Phyllospora comosa* germlings with rounded shape (%) from different populations (Black Head and Palm Beach) and under three MHW scenarios (Ambient, strong and extreme MHW) after 9, 16, 23 and 30 days. |
| 21-22 | Table S9: Summary of *Phyllospora comosa* juvenile experiment results. |
| 23 | Table S10: Differences in *Phyllospora comosa* juvenile survival (0-1) from different populations (Bonny Hills, Black Head, Palm Beach, Cronulla) and under three MHW scenarios (Ambient, strong and extreme MHW). |
| 24 | Table S11: Differences in *Phyllospora comosa* juvenile relative growth rate (RGR %d^-1^) from different populations (Bonny Hills, Black Head, Palm Beach, Cronulla) and under ambient and strong MHW scenarios. |
| 25 | Table S12: Differences in *Phyllospora comosa* juvenile Fv/Fm from different populations (Bonny Hills, Black Head, Palm Beach, Cronulla) and under three MHW scenarios (Ambient, strong and extreme MHW). |
| 26 | Table S13: Differences in *Phyllospora comosa* juvenile structural integrity loss (0-4) from different populations (Bonny Hills, Black Head, Palm Beach, Cronulla) and under three MHW scenarios (Ambient, strong and extreme MHW). |
| 27 | Table S14: Differences in *Phyllospora comosa* juvenile fouling level (0-4) from different populations (Bonny Hills, Black Head, Palm Beach, Cronulla) and under three MHW scenarios (Ambient, strong and extreme MHW). |
| 28 | Table S15: Differences in *Phyllospora comosa* juvenile bleaching level (0-4) from different populations (Bonny Hills, Black Head, Palm Beach, Cronulla) and under three MHW scenarios (Ambient, strong and extreme MHW). |
| 29 | Table S16: Differences in *Phyllospora comosa* juvenile necrosis (0-4) from different populations (Bonny Hills, Black Head, Palm Beach, Cronulla) and under three MHW scenarios (Ambient, strong and extreme MHW). |
| 30 | Table S17: Differences in *Phyllospora comosa* juvenile green colouration (0-4) from different populations (Bonny Hills, Black Head, Palm Beach, Cronulla) and under three MHW scenarios (Ambient, strong and extreme MHW). |
| 31 | Table S18: Differences in *Phyllospora comosa* juvenile C:N ratio, and tissue carbon (C) and nitrogen (N, %) from different populations (Bonny Hills, Black Head, Palm Beach, Cronulla) and under ambient and strong MHW scenarios. |
| 32 | Table S19: Differences in *Phyllospora comosa* juvenile pigment content (chlorophyll-a, chlorophyll-c and fucoxanthin, mg g^-1^) from different populations (Bonny Hills, Black Head, Palm Beach, Cronulla) and under ambient and strong MHW scenarios. |

**Figures**

**
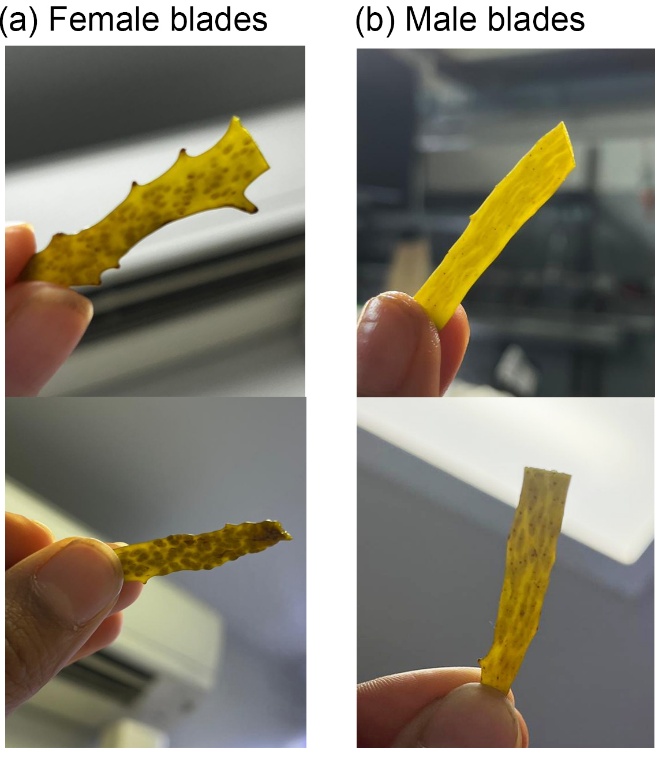
**

**Figure S1: Visual differences between (a) female and (b) male *Phyllospora comosa* blades.** Female blades tend to have rounded conceptacles, which are darker than the elongated male conceptacles.

**
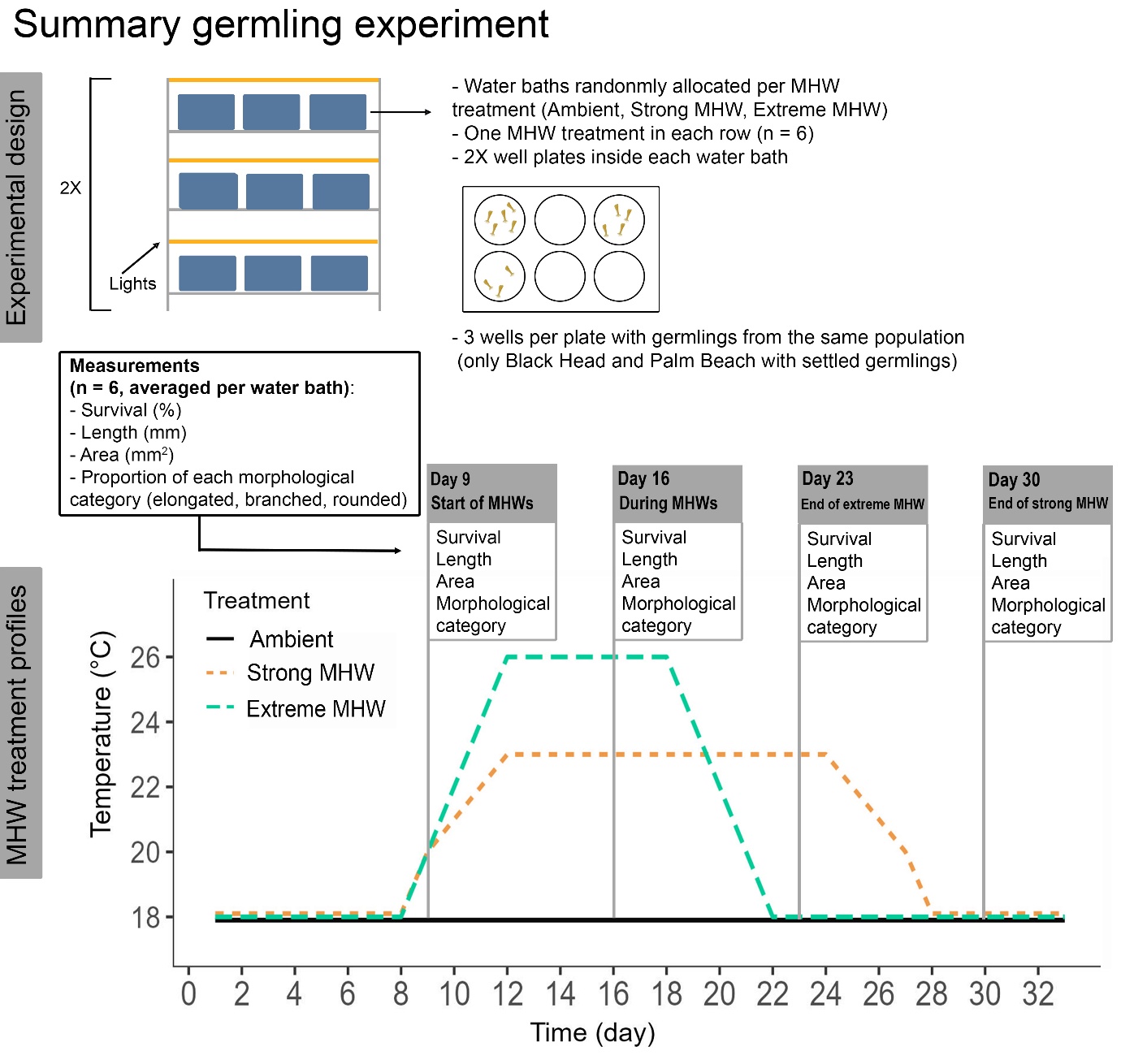
Figure S2: Summary of *Phyllospora comosa* germling experiment**, showing the experimental design, MHW treatment profiles and measurements conducted.


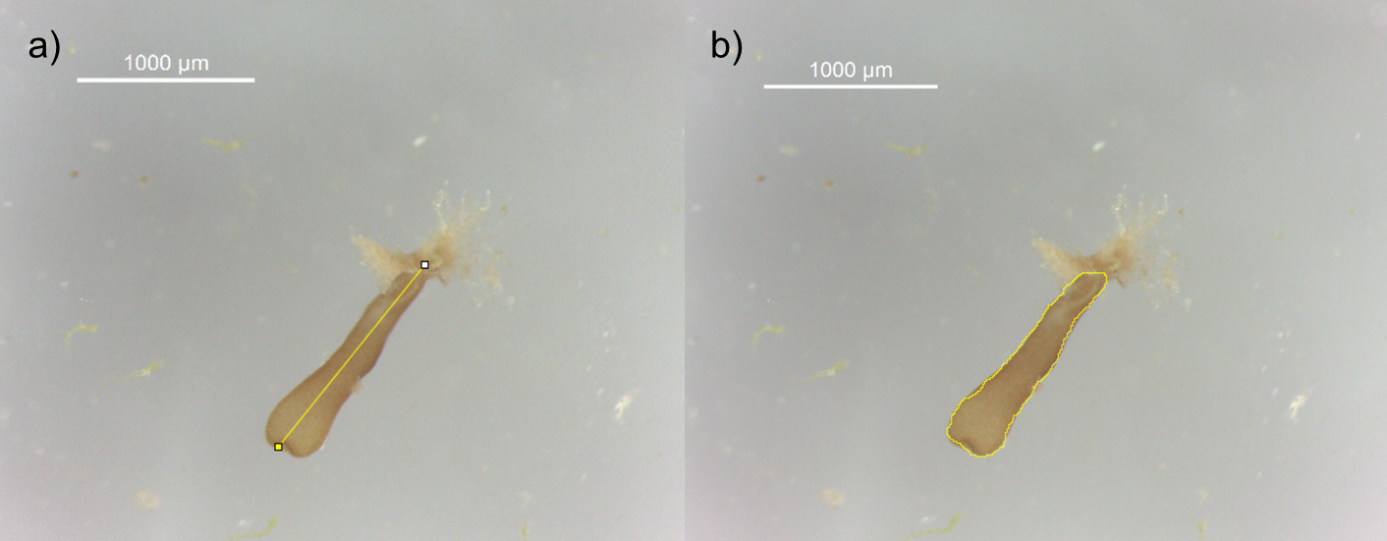


**Figure S3: Phyllospora comosa germling measurements to record a) length (mm) and b) area (mm^2^).**


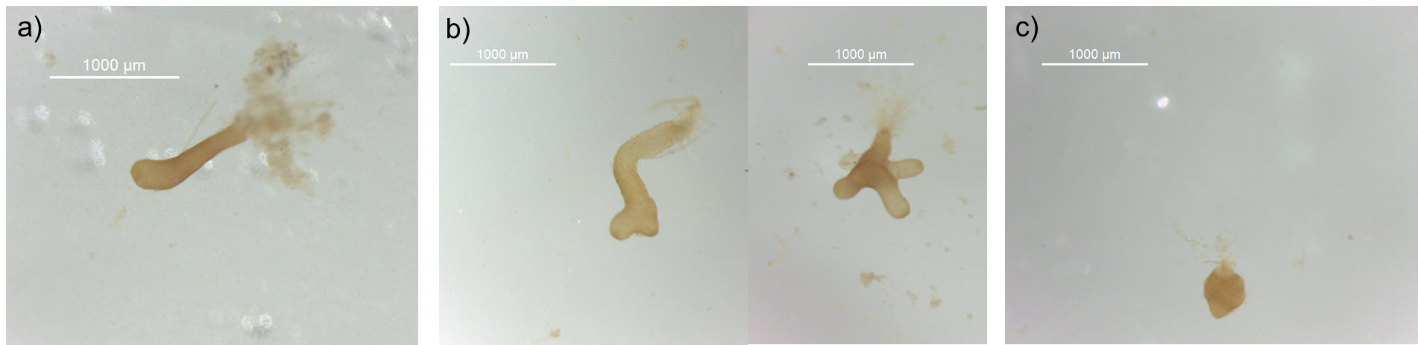


**Figure S4: Phyllospora comosa germlings shape categories a) elongated, b) branched, c) rounded.**

**
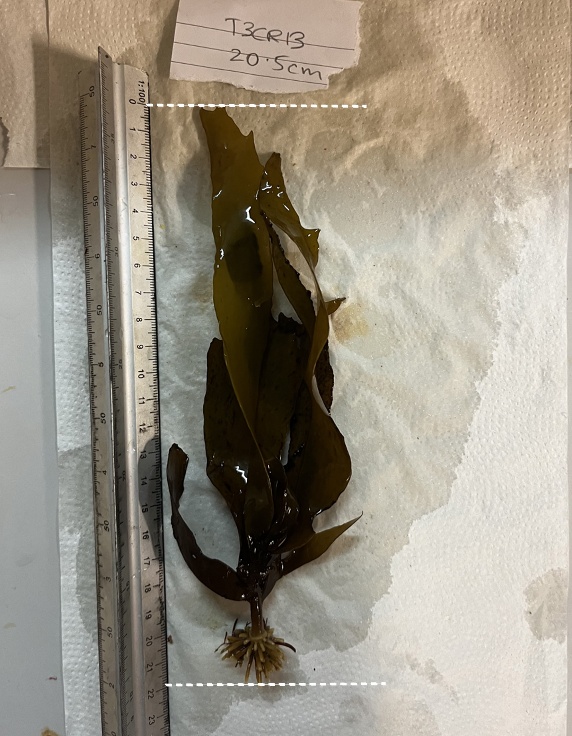
**

**Figure S5: Phyllospora comosa juvenile full individual length example.** White dashed lines indicate the limits of the measurement

**
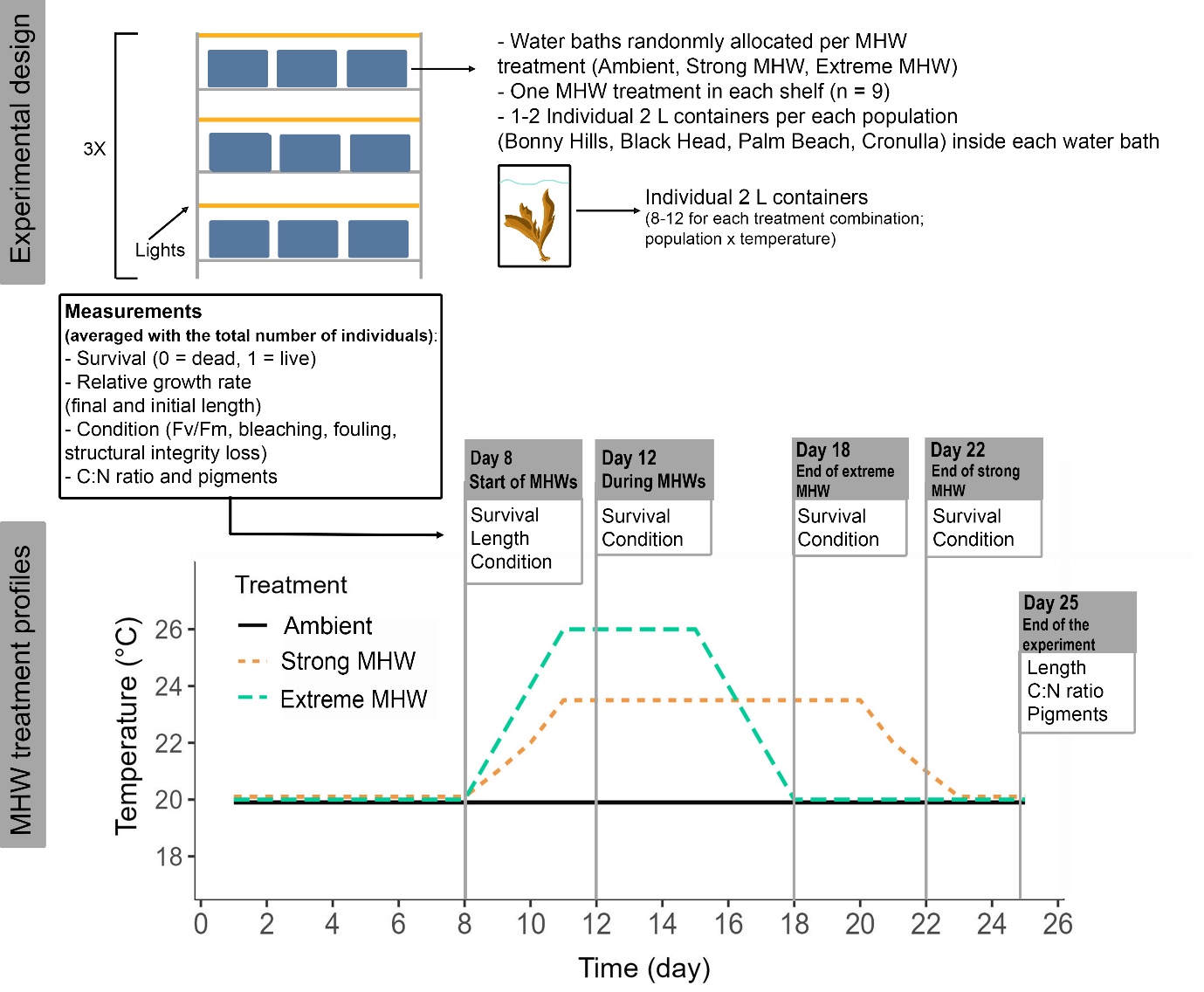
**

**Figure S6: Summary of *Phyllospora comosa* juvenile experiment**, showing the experimental design, MHW treatment profiles and measurements conducted.


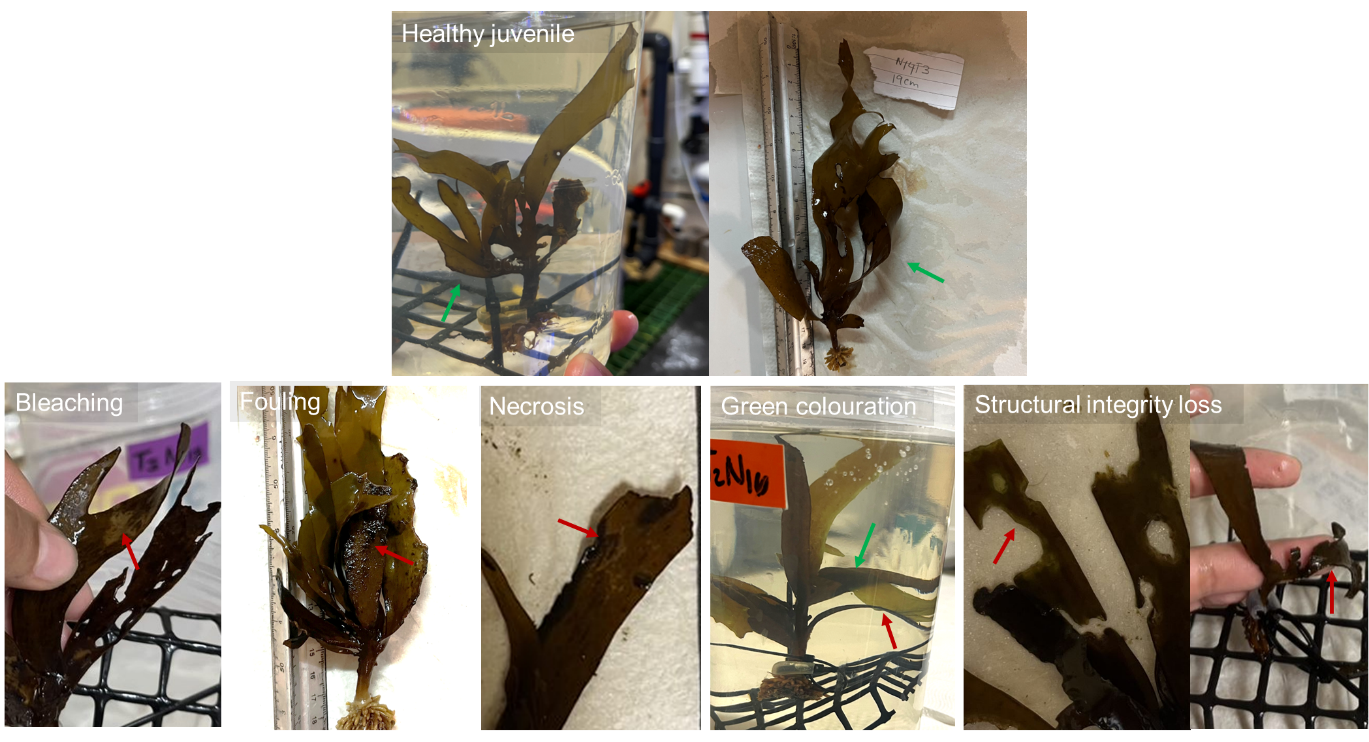


**Figure S7: Example of condition metrics recorded in *Phyllospora comosa* juveniles.** Values for condition ranged from 0-4, corresponding to 0%, < 25%, 30-40%, 60-70%, and > 80 % of the juvenile tissue. Red arrows indicate affected tissue, while green arrows indicate healthy tissue.

*
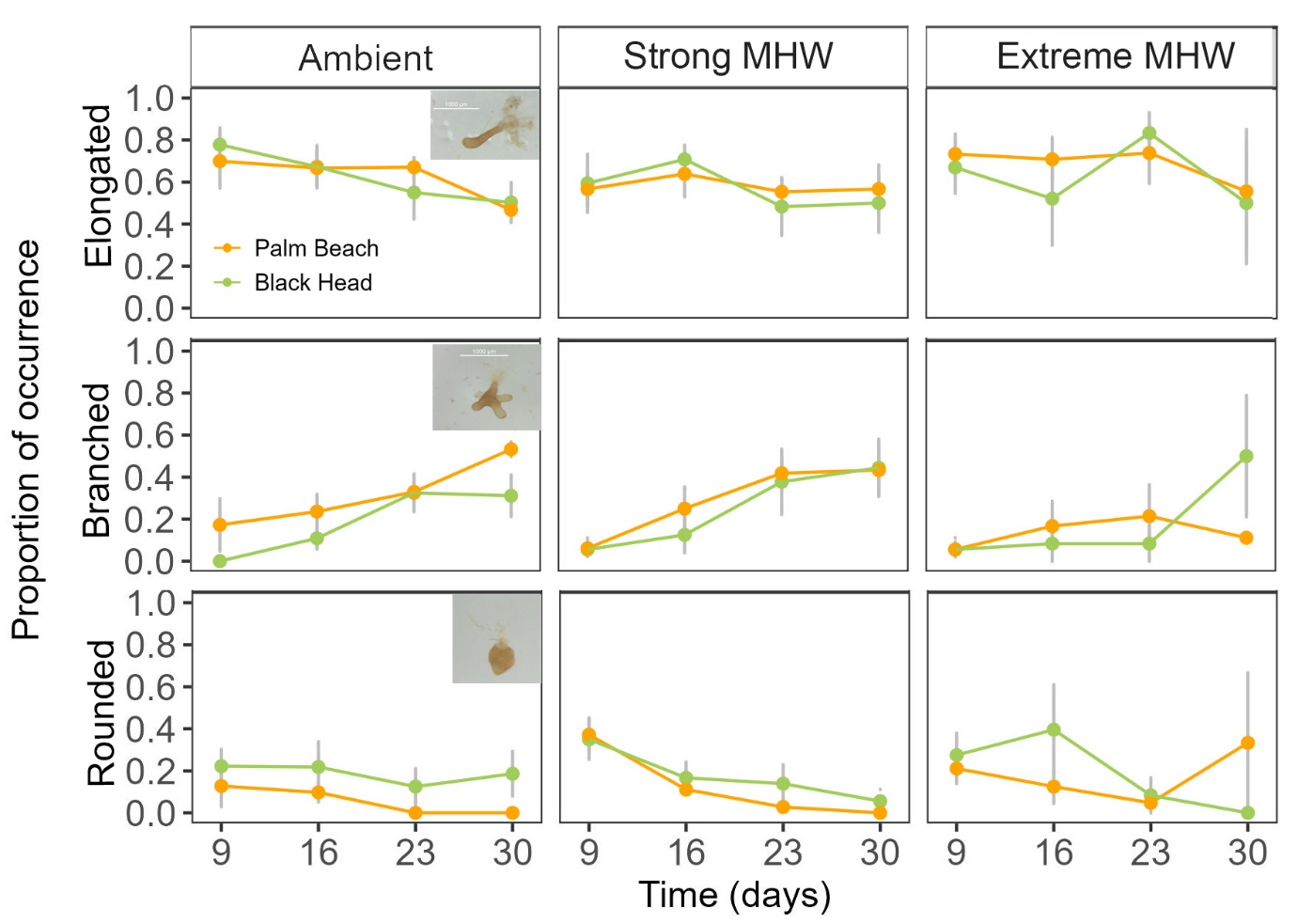
*

**Figure S8: Proportion of Phyllospora comosa germlings with different shapes (n = 6)** out of the total individuals belonging to each population, MHW treatment and day combination. Strong marine heatwave reached 23.5°C, and extreme MHW reached 26°C. Ambient treatment was maintained at 18°C as a control.


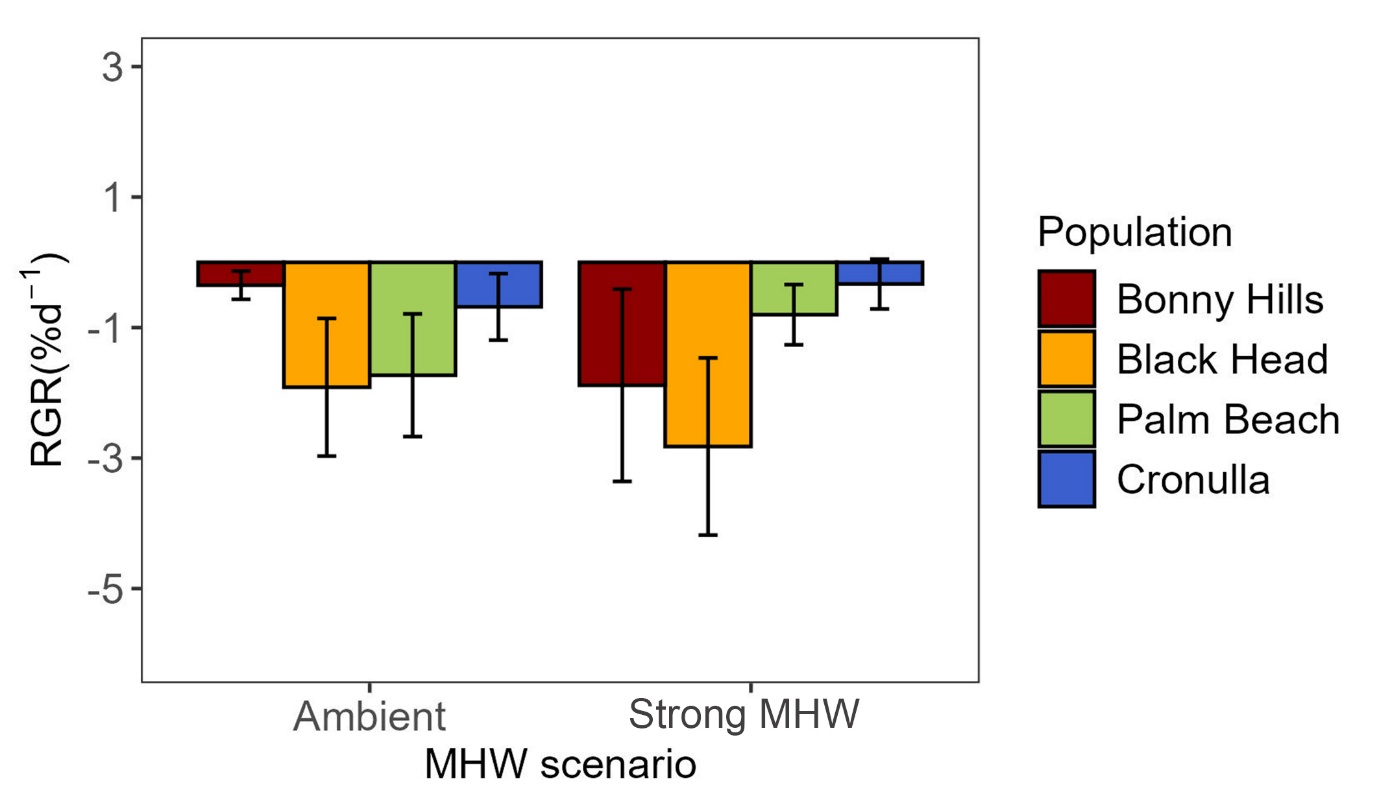


**Figure S9: Relative growth rate (RGR %d^-1^; mean +\- SE; n = 8-12) in Phyllospora comosa juveniles from four populations along New South Wales** exposed to a strong MHW (max. temp. = 23.5°C), an extreme MHW (max. temp. = 26°C) and ambient conditions (20°C).


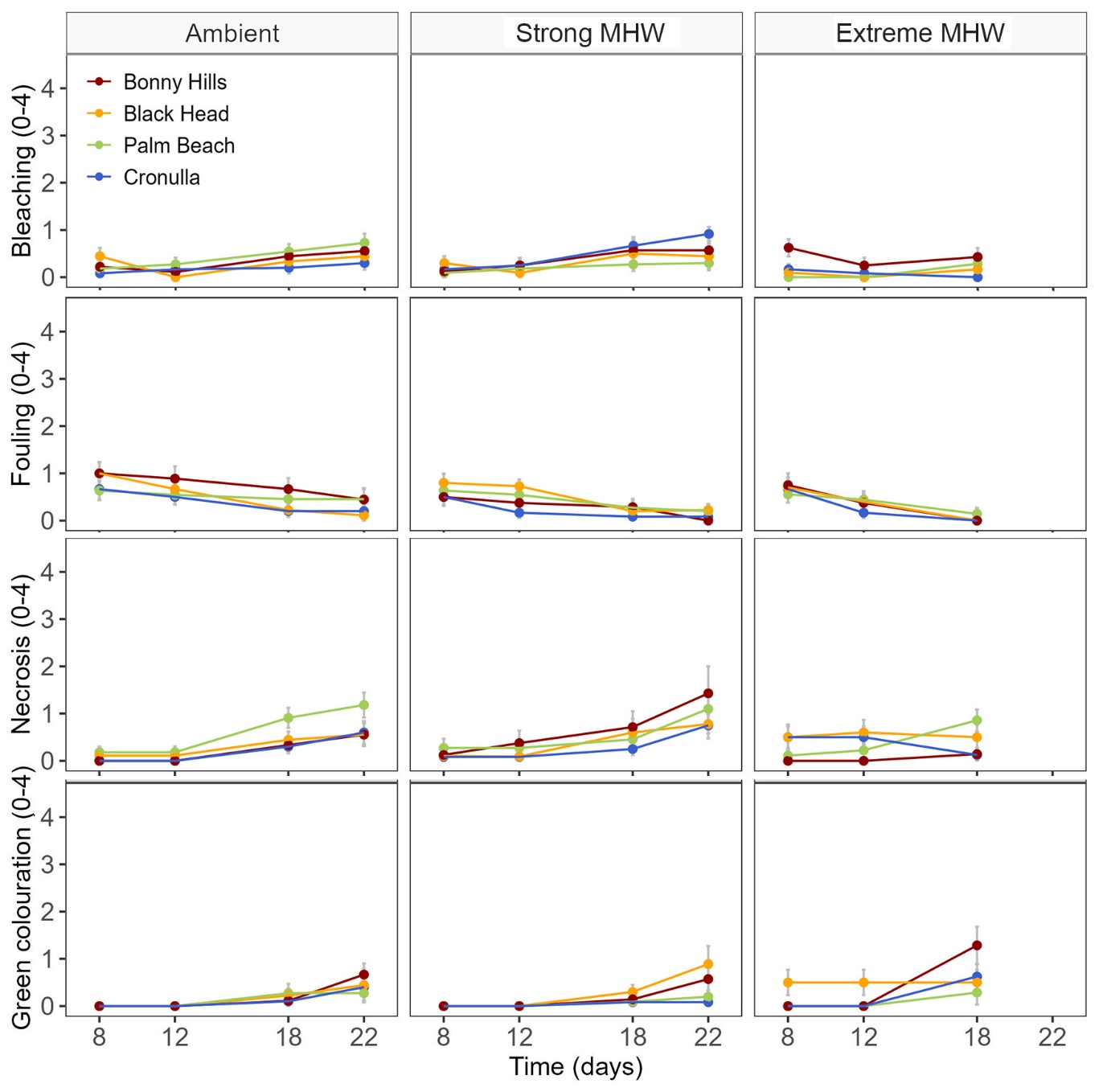


**Figure S10: Phyllospora comosa juvenile condition metrics: bleaching, fouling, necrosis and green colouration (mean, +\- SE, n = 6) under experimental MHW scenarios**. MHW1 corresponds to a strong MHW (max. temp. = 23°C), and MHW2 corresponds to an extreme MHW (max. temp. = 26°C). Ambient treatment was maintained at 20°C as a control. Values for condition ranged from 0-4 corresponding to 0%, < 25%, 30-40%, 60-70%, and > 80 %.

**Tables**

**Table S1: Summary of strong and severe MHW events in four sites along New South Wales.** Data for historical events were obtained from the Marine Heatwave Tracker climatology and thresholds database (<https://www.marineheatwaves.org/>), utilising the nearest coordinates to each study site. No extreme MHW events have been recorded in these sites between 1982 and 2025. Intensity levels (strong and severe) were defined as how many times the highest temperature of a MHW surpasses the 90th percentile threshold of the climatology of a site (2x = Strong, 3x = Severe; based on average site temperatures)

| Site | # Strong MHWs | Average strong MHW duration (days) | Maximum intensity strong MHW (°C) | # Severe MHWs | Average severe MHW duration (Days) | Maximum intensity severe MHW (°C) |
| --- | --- | --- | --- | --- | --- | --- |
| Bonny Hills  (31°35'39.7"S 152°50'36.5" E) | 34 | 15 | 3.4 | 0 |  |  |
| Black Head (32°04'11.8"S 152°32'45.7" E) | 30 | 13 | 3.68 | 8 | 17 | 4.25 |
| Palm Beach (33°35'58.1"S 151°19'41.0" E) | 37 | 20 | 3.67 | 10 | 19 | 3.77 |
| Cronulla (34°04'13.7"S 151°09'24.8" E) | 42 | 22 | 4.19 | 4 | 47 | 4.13 |

**Table S2: Summary of *Phyllospora comosa* germling experiment results.** Values provided correspond to mean **±** SE

| Site | MHW treatment | Day | Survival (%) | | | Length (mm) | | | Area (mm^2^) | | | Proportion elongated | | | Proportion branched | | | Proportion rounded | | |
| --- | --- | --- | --- | --- | --- | --- | --- | --- | --- | --- | --- | --- | --- | --- | --- | --- | --- | --- | --- | --- |
| Black Head (warm-edge) | Ambient | 9 | 100.00 | **±** | 0.00 | 0.51 | **±** | 0.04 | 0.07 | **±** | 0.01 | 0.78 | **±** | 0.08 | 0.00 | **±** | 0.00 | 0.22 | **±** | 0.08 |
|  |  | 16 | 97.62 | **±** | 2.38 | 0.73 | **±** | 0.06 | 0.15 | **±** | 0.02 | 0.67 | **±** | 0.10 | 0.11 | **±** | 0.05 | 0.22 | **±** | 0.12 |
|  |  | 23 | 94.84 | **±** | 3.28 | 0.83 | **±** | 0.07 | 0.20 | **±** | 0.03 | 0.55 | **±** | 0.13 | 0.33 | **±** | 0.09 | 0.13 | **±** | 0.09 |
|  |  | 30 | 85.12 | **±** | 8.17 | 0.95 | **±** | 0.10 | 0.25 | **±** | 0.02 | 0.50 | **±** | 0.09 | 0.31 | **±** | 0.10 | 0.19 | **±** | 0.11 |
|  | Strong MHW | 9 | 100.00 | **±** | 0.00 | 0.50 | **±** | 0.03 | 0.07 | **±** | 0.01 | 0.59 | **±** | 0.14 | 0.06 | **±** | 0.06 | 0.35 | **±** | 0.09 |
|  |  | 16 | 93.33 | **±** | 4.22 | 0.85 | **±** | 0.07 | 0.18 | **±** | 0.01 | 0.71 | **±** | 0.07 | 0.13 | **±** | 0.09 | 0.17 | **±** | 0.07 |
|  |  | 23 | 93.33 | **±** | 4.22 | 1.06 | **±** | 0.08 | 0.26 | **±** | 0.02 | 0.48 | **±** | 0.14 | 0.38 | **±** | 0.16 | 0.14 | **±** | 0.09 |
|  |  | 30 | 90.00 | **±** | 6.83 | 1.24 | **±** | 0.07 | 0.33 | **±** | 0.01 | 0.50 | **±** | 0.14 | 0.44 | **±** | 0.14 | 0.06 | **±** | 0.06 |
|  | Extreme MHW | 9 | 100.00 | **±** | 0.00 | 0.54 | **±** | 0.03 | 0.08 | **±** | 0.00 | 0.67 | **±** | 0.12 | 0.06 | **±** | 0.06 | 0.28 | **±** | 0.10 |
|  |  | 16 | 61.39 | **±** | 10.46 | 0.64 | **±** | 0.06 | 0.11 | **±** | 0.01 | 0.52 | **±** | 0.22 | 0.08 | **±** | 0.08 | 0.40 | **±** | 0.21 |
|  |  | 23 | 33.61 | **±** | 12.10 | 0.83 | **±** | 0.04 | 0.17 | **±** | 0.01 | 0.83 | **±** | 0.10 | 0.08 | **±** | 0.08 | 0.08 | **±** | 0.08 |
|  |  | 30 | 33.61 | **±** | 12.10 | 1.05 | **±** | 0.09 | 0.25 | **±** | 0.02 | 0.50 | **±** | 0.29 | 0.50 | **±** | 0.29 | 0.00 | **±** | 0.00 |
| Palm Beach (central) | Ambient | 9 | 100.00 | **±** | 0.00 | 0.51 | **±** | 0.03 | 0.07 | **±** | 0.00 | 0.70 | **±** | 0.13 | 0.17 | **±** | 0.12 | 0.13 | **±** | 0.10 |
|  |  | 16 | 100.00 | **±** | 0.00 | 0.69 | **±** | 0.04 | 0.13 | **±** | 0.01 | 0.67 | **±** | 0.09 | 0.24 | **±** | 0.08 | 0.10 | **±** | 0.05 |
|  |  | 23 | 97.78 | **±** | 2.22 | 0.84 | **±** | 0.06 | 0.18 | **±** | 0.01 | 0.67 | **±** | 0.05 | 0.33 | **±** | 0.05 | 0.00 | **±** | 0.00 |
|  |  | 30 | 94.58 | **±** | 3.56 | 0.98 | **±** | 0.06 | 0.23 | **±** | 0.02 | 0.47 | **±** | 0.03 | 0.53 | **±** | 0.03 | 0.00 | **±** | 0.00 |
|  | Strong MHW | 9 | 100.00 | **±** | 0.00 | 0.46 | **±** | 0.03 | 0.06 | **±** | 0.00 | 0.57 | **±** | 0.08 | 0.06 | **±** | 0.04 | 0.37 | **±** | 0.08 |
|  |  | 16 | 99.17 | **±** | 0.83 | 0.74 | **±** | 0.05 | 0.15 | **±** | 0.01 | 0.64 | **±** | 0.11 | 0.25 | **±** | 0.10 | 0.11 | **±** | 0.11 |
|  |  | 23 | 91.29 | **±** | 4.70 | 0.89 | **±** | 0.05 | 0.20 | **±** | 0.01 | 0.55 | **±** | 0.06 | 0.42 | **±** | 0.07 | 0.03 | **±** | 0.03 |
|  |  | 30 | 91.29 | **±** | 4.70 | 1.05 | **±** | 0.06 | 0.27 | **±** | 0.02 | 0.57 | **±** | 0.12 | 0.43 | **±** | 0.12 | 0.00 | **±** | 0.00 |
|  | Extreme MHW | 9 | 100.00 | **±** | 0.00 | 0.47 | **±** | 0.01 | 0.07 | **±** | 0.00 | 0.73 | **±** | 0.10 | 0.06 | **±** | 0.04 | 0.21 | **±** | 0.07 |
|  |  | 16 | 50.32 | **±** | 15.18 | 0.65 | **±** | 0.04 | 0.11 | **±** | 0.01 | 0.71 | **±** | 0.10 | 0.17 | **±** | 0.12 | 0.13 | **±** | 0.08 |
|  |  | 23 | 23.89 | **±** | 15.88 | 0.74 | **±** | 0.03 | 0.14 | **±** | 0.01 | 0.74 | **±** | 0.14 | 0.21 | **±** | 0.15 | 0.05 | **±** | 0.05 |
|  |  | 30 | 20.40 | **±** | 13.58 | 0.74 | **±** | 0.09 | 0.17 | **±** | 0.02 | 0.56 | **±** | 0.29 | 0.11 | **±** | 0.11 | 0.33 | **±** | 0.33 |

**Table S3: Differences in *Phyllospora comosa* germling survival (%) from different populations (Black Head and Palm Beach) and under three MHW scenarios (Ambient, strong and extreme MHW) after 9, 16, 23 and 30 days** based on Linear Mixed Models (LMMs) with water bath as a random factor. Differences were added for significant effects and only for significant interactions in case they occurred. Surv was used as an abbreviation for survival.

| Response | Factor | Differences | | χ^2^ | df | p-value |
| --- | --- | --- | --- | --- | --- | --- |
| Survival | Population |  |  | 0.22 | 1 | 0.64 |
|  | MHW |  |  | 57.67 | 2 | **< 0.001** |
|  | Day |  |  | 92.35 | 3 | **< 0.001** |
|  | Population:MHW |  |  | 4.42 | 2 | 0.11 |
|  | Population:Day |  |  | 0.19 | 3 | 0.98 |
|  | MHW:Day | Day 16 - | Surv_strong_ < Surv_ambient_ | 98.27 | 6 | **< 0.001** |
|  |  |  | Surv_extreme_ < Surv_strong_ |  |  |  |
|  |  | Day 23 - | Surv_extreme_ < Surv_ambient_ |  |  |  |
|  |  |  | Surv_extreme_ < Surv_strong_ |  |  |  |
|  |  | Day 30 - | Surv_extreme_ < Surv_ambient_ |  |  |  |
|  |  |  | Surv_extreme_ < Surv_strong_ |  |  |  |
|  |  | Surv_extreme_ - | Day 9 > Day 16 |  |  |  |
|  |  |  | Day 9 > Day 23 |  |  |  |
|  |  |  | Day 9 > Day 30 |  |  |  |
|  |  |  | Day 16 < Day 23 |  |  |  |
|  |  |  | Day 16 < Day 30 |  |  |  |
|  | Population:MHW:Day |  |  | 2.27 | 6 | 0.89 |

**Table S4: Differences in *Phyllospora comosa* germling area (mm^2^) from different populations (Black Head and Palm Beach) and under three MHW scenarios (Ambient, strong and extreme MHW) after 9, 16, 23 and 30 days** based on Linear Mixed Models (LMMs) with water bath as a random factor. Differences were added for significant effects and only for significant interactions in case they occurred.

| Response | Factor | Differences | | χ^2^ | df | p-value |
| --- | --- | --- | --- | --- | --- | --- |
| Area | Population |  |  | 29.66 | 1 | **< 0.001** |
|  | MHW |  |  | 12.06 | 2 | **< 0.01** |
|  | Day |  |  | 882.63 | 3 | **< 0.001** |
|  | Population:MHW |  |  | 3.07 | 2 | 0.22 |
|  | Population:Day | Day 23 - | Area_Black Head_ > Area_Black Head_ | 13.80 | 3 | **< 0.01** |
|  |  | Day 30 - | Area_Black Head_ > Area_Palm Beach_ |  |  |  |
|  | MHW:Day | Day 16 - | Area_extreme_ < Area_strong_ | 50.99 | 6 | **< 0.001** |
|  |  | Day 23 - | Area_extreme_ < Area_ambient_ < Area_strong_ |  |  |  |
|  |  | Day 30 - | Area_extreme_ < Area_ambient_ < Area_strong_ |  |  |  |
|  | Population:MHW:Day |  |  | 8.52 | 6 | 0.20 |

**Table S5: Differences in *Phyllospora comosa* germling length (mm) from different populations (Black Head and Palm Beach) and under three MHW scenarios (Ambient, strong and extreme MHW) after 9, 16, 23 and 30 days** based on Linear Mixed Models (LMMs) with water bath as a random factor. Differences were added for significant effects and only for significant interactions in case they occurred.

| Response | Factor | Differences | | χ^2^ | df | p-value |
| --- | --- | --- | --- | --- | --- | --- |
| Length | Population |  |  | 19.35 | 1 | **< 0.001** |
|  | MHW |  |  | 5.26 | 2 | 0.07 |
|  | Day |  |  | 502.43 | 3 | **< 0.001** |
|  | Population:MHW | Black Head - | Length_Strong_ > Length_ambient_ | 13.16 | 2 | **< 0.01** |
|  |  |  | Length_Strong_ > Length_extreme_ |  |  |  |
|  |  | Palm Beach - | Length_Strong_ > Length_extreme_ |  |  |  |
|  |  | MHW1 - | Length_Black Head_> Length_Palm Beach_ |  |  |  |
|  |  | MHW2 - | Length_Black Head_> Length_Palm Beach_ |  |  |  |
|  | Population:Day |  |  | 4.89 | 3 | 0.18 |
|  | MHW:Day | Day 16 - | Length_Strong_ > Length_extreme_ | 29.23 | 6 | **< 0.001** |
|  |  | Day 23 - | Length_Strong_> Length_extreme_ |  |  |  |
|  |  | Day 30 - | Length_Strong_ > Length_extreme_ |  |  |  |
|  |  | Day 30 - | Length_Strong_> Length_ambient_ |  |  |  |
|  | Population:MHW:Day |  |  | 11.80 | 6 | 0.07 |

**Table S6: Differences in *Phyllospora comosa* germlings with elongated shape (%) from different populations (Black Head and Palm Beach) and under three MHW scenarios (Ambient, strong and extreme MHW) after 9, 16, 23 and 30 days** based on Linear Mixed Models (LMMs) with water bath as a random factor.

| Response | Factor | χ^2^ | df | p-value |
| --- | --- | --- | --- | --- |
| Elongated | Population | 0.13 | 1 | 0.72 |
|  | MHW | 0.86 | 2 | 0.65 |
|  | Day | 7.01 | 3 | 0.07 |
|  | Population:MHW | 0.25 | 2 | 0.88 |
|  | Population:Day | 0.31 | 3 | 0.96 |
|  | MHW:Day | 6.15 | 6 | 0.41 |
|  | Population:MHW:Day | 2.39 | 6 | 0.88 |

**Table S7: Differences in *Phyllospora comosa* germlings with branched shape (%) from different populations (Black Head and Palm Beach) and under three MHW scenarios (Ambient, strong and extreme MHW) after 9, 16, 23 and 30 days** based on Linear Mixed Models (LMMs) with water bath as a random factor. Differences were added for significant effects.

| Response | Factor | Differences |  | χ^2^ | df | p-value |
| --- | --- | --- | --- | --- | --- | --- |
| Branched | Population |  |  | 1.93 | 1 | 0.17 |
|  | MHW |  |  | 3.48 | 2 | 0.18 |
|  | Day | Day 9 < Day 16 < Day 23 | | 41.14 | 3 | **< 0.001** |
|  |  | Day 9 < Day 16 < Day 30 | |  |  |  |
|  | Population:MHW |  |  | 2.70 | 2 | 0.26 |
|  | Population:Day |  |  | 1.20 | 3 | 0.75 |
|  | MHW:Day |  |  | 3.55 | 6 | 0.74 |
|  | Population:MHW:Day |  |  | 6.58 | 6 | 0.36 |

**Table S8: Differences in *Phyllospora comosa* germlings with rounded shape (%) from different populations (Black Head and Palm Beach) and under three MHW scenarios (Ambient, strong and extreme MHW) after 9, 16, 23 and 30 days** based on Linear Mixed Models (LMMs) with water bath as a random factor. Differences were added for significant effects.

| Response | Factor | Differences | χ^2^ | df | p-value |
| --- | --- | --- | --- | --- | --- |
| Rounded | Population |  | 5.68 | 1 | **0.02** |
|  | MHW |  | 0.55 | 2 | 0.76 |
|  | Day | Day 9 > Day 23 | 22.14 | 3 | **< 0.001** |
|  |  | Day 9 > Day 30 |  |  |  |
|  |  | Day 16 > Day 23 |  |  |  |
|  | Population:MHW |  | 1.89 | 2 | 0.39 |
|  | Population:Day |  | 1.85 | 3 | 0.60 |
|  | MHW:Day |  | 9.57 | 6 | 0.14 |
|  | Population:MHW:Day |  | 9.83 | 6 | 0.13 |

**Table S9: Summary of *Phyllospora comosa* juvenile experiment results.** Values provided correspond to mean **±** SE

| Site | MHW treatment | Day | Survival (0 or 1) | | | Fv/Fm | | | Structural integrity loss (0-4) | | | Bleaching  (0-4) | | | Fouling (0-4) | | | Necrosis  (0-4) | | | Green colouration (0-4) | | | RGR (%d-1) | | | Tissue Carbon | | | Tissue Nitrogen | | | C:N ratio | | | Chlorophyll-a | | | Chlorophyll-c | | | Fucoxanthin | | |
| --- | --- | --- | --- | --- | --- | --- | --- | --- | --- | --- | --- | --- | --- | --- | --- | --- | --- | --- | --- | --- | --- | --- | --- | --- | --- | --- | --- | --- | --- | --- | --- | --- | --- | --- | --- | --- | --- | --- | --- | --- | --- | --- | --- | --- |
| Bonny Hills (warm-edge) | Ambient | 8 | 1.00 | **±** | 0.00 | 0.40 | **±** | 0.06 | 0.22 | **±** | 0.15 | 0.22 | **±** | 0.15 | 1.00 | **±** | 0.24 | 0.00 | **±** | 0.00 | 0.00 | **±** | 0.00 |  | **±** |  |  | **±** |  |  | **±** |  |  | **±** |  |  | **±** |  |  | **±** |  |  | **±** |  |
|  |  | 12 | 1.00 | **±** | 0.00 | 0.42 | **±** | 0.06 | 0.33 | **±** | 0.17 | 0.11 | **±** | 0.11 | 0.89 | **±** | 0.26 | 0.00 | **±** | 0.00 | 0.00 | **±** | 0.00 |  | **±** |  |  | **±** |  |  | **±** |  |  | **±** |  |  | **±** |  |  | **±** |  |  | **±** |  |
|  |  | 18 | 1.00 | **±** | 0.00 | 0.32 | **±** | 0.03 | 0.56 | **±** | 0.18 | 0.44 | **±** | 0.18 | 0.67 | **±** | 0.24 | 0.33 | **±** | 0.17 | 0.11 | **±** | 0.11 |  | **±** |  |  | **±** |  |  | **±** |  |  | **±** |  |  | **±** |  |  | **±** |  |  | **±** |  |
|  |  | 22 | 1.00 | **±** | 0.00 | 0.34 | **±** | 0.03 | 0.78 | **±** | 0.22 | 0.56 | **±** | 0.18 | 0.44 | **±** | 0.24 | 0.56 | **±** | 0.24 | 0.67 | **±** | 0.24 | -0.35 | **±** | 0.22 | 31.73 | **±** | 0.69 | 0.92 | **±** | 0.05 | 34.87 | **±** | 1.93 | 0.008 | **±** | 0.002 | 0.003 | **±** | 0.0002 | 0.003 | **±** | 0.001 |
|  | Strong MHW | 8 | 1.00 | **±** | 0.00 | 0.34 | **±** | 0.04 | 0.38 | **±** | 0.18 | 0.13 | **±** | 0.13 | 0.50 | **±** | 0.19 | 0.13 | **±** | 0.13 | 0.00 | **±** | 0.00 |  | **±** |  |  | **±** |  |  | **±** |  |  | **±** |  |  | **±** |  |  | **±** |  |  | **±** |  |
|  |  | 12 | 1.00 | **±** | 0.00 | 0.38 | **±** | 0.05 | 1.00 | **±** | 0.46 | 0.25 | **±** | 0.16 | 0.38 | **±** | 0.18 | 0.38 | **±** | 0.26 | 0.00 | **±** | 0.00 |  | **±** |  |  | **±** |  |  | **±** |  |  | **±** |  |  | **±** |  |  | **±** |  |  | **±** |  |
|  |  | 18 | 0.75 | **±** | 0.16 | 0.26 | **±** | 0.06 | 1.00 | **±** | 0.50 | 0.57 | **±** | 0.28 | 0.29 | **±** | 0.17 | 0.71 | **±** | 0.34 | 0.14 | **±** | 0.13 |  | **±** |  |  | **±** |  |  | **±** |  |  | **±** |  |  | **±** |  |  | **±** |  |  | **±** |  |
|  |  | 22 | 0.75 | **±** | 0.16 | 0.19 | **±** | 0.06 | 1.71 | **±** | 0.57 | 0.57 | **±** | 0.19 | 0.00 | **±** | 0.00 | 1.43 | **±** | 0.57 | 0.57 | **±** | 0.40 | -1.88 | **±** | 1.47 | 32.30 | **±** | 0.56 | 0.75 | **±** | 0.07 | 45.02 | **±** | 4.78 | 0.009 | **±** | 0.002 | 0.004 | **±** | 0.0003 | 0.003 | **±** | 0.001 |
|  | Extreme MHW | 8 | 1.00 | **±** | 0.00 | 0.28 | **±** | 0.04 | 0.25 | **±** | 0.16 | 0.63 | **±** | 0.18 | 0.75 | **±** | 0.25 | 0.00 | **±** | 0.00 | 0.00 | **±** | 0.00 |  | **±** |  |  | **±** |  |  | **±** |  |  | **±** |  |  | **±** |  |  | **±** |  |  | **±** |  |
|  |  | 12 | 0.88 | **±** | 0.13 | 0.39 | **±** | 0.04 | 0.75 | **±** | 0.49 | 0.25 | **±** | 0.16 | 0.38 | **±** | 0.18 | 0.00 | **±** | 0.00 | 0.00 | **±** | 0.00 |  | **±** |  |  | **±** |  |  | **±** |  |  | **±** |  |  | **±** |  |  | **±** |  |  | **±** |  |
|  |  | 18 | 0.13 | **±** | 0.13 | 0.09 | **±** | 0.04 | 3.43 | **±** | 0.53 | 0.43 | **±** | 0.19 | 0.00 | **±** | 0.00 | 0.14 | **±** | 0.13 | 1.29 | **±** | 0.39 | -3.05 | **±** | 0.98 |  | **±** |  |  | **±** |  |  | **±** |  |  | **±** |  |  | **±** |  |  | **±** |  |
| Black Head (warm-edge) | Ambient | 8 | 1.00 | **±** | 0.00 | 0.32 | **±** | 0.05 | 0.56 | **±** | 0.18 | 0.44 | **±** | 0.18 | 1.00 | **±** | 0.24 | 0.11 | **±** | 0.11 | 0.00 | **±** | 0.00 |  | **±** |  |  | **±** |  |  | **±** |  |  | **±** |  |  | **±** |  |  | **±** |  |  | **±** |  |
|  |  | 12 | 1.00 | **±** | 0.00 | 0.39 | **±** | 0.04 | 1.22 | **±** | 0.40 | 0.00 | **±** | 0.00 | 0.67 | **±** | 0.24 | 0.11 | **±** | 0.11 | 0.00 | **±** | 0.00 |  | **±** |  |  | **±** |  |  | **±** |  |  | **±** |  |  | **±** |  |  | **±** |  |  | **±** |  |
|  |  | 18 | 0.89 | **±** | 0.11 | 0.26 | **±** | 0.04 | 1.33 | **±** | 0.47 | 0.33 | **±** | 0.17 | 0.22 | **±** | 0.15 | 0.44 | **±** | 0.18 | 0.22 | **±** | 0.15 |  | **±** |  |  | **±** |  |  | **±** |  |  | **±** |  |  | **±** |  |  | **±** |  |  | **±** |  |
|  |  | 22 | 0.78 | **±** | 0.15 | 0.28 | **±** | 0.06 | 1.44 | **±** | 0.53 | 0.44 | **±** | 0.18 | 0.11 | **±** | 0.11 | 0.56 | **±** | 0.24 | 0.44 | **±** | 0.34 | -1.91 | **±** | 1.05 | 31.42 | **±** | 1.11 | 0.85 | **±** | 0.05 | 37.81 | **±** | 2.99 | 0.008 | **±** | 0.001 | 0.003 | **±** | 0.0001 | 0.003 | **±** | 0.001 |
|  | Strong MHW | 8 | 1.00 | **±** | 0.00 | 0.32 | **±** | 0.05 | 0.30 | **±** | 0.15 | 0.30 | **±** | 0.15 | 0.80 | **±** | 0.19 | 0.10 | **±** | 0.10 | 0.00 | **±** | 0.00 |  | **±** |  |  | **±** |  |  | **±** |  |  | **±** |  |  | **±** |  |  | **±** |  |  | **±** |  |
|  |  | 12 | 1.00 | **±** | 0.00 | 0.39 | **±** | 0.05 | 0.55 | **±** | 0.28 | 0.09 | **±** | 0.09 | 0.73 | **±** | 0.14 | 0.09 | **±** | 0.09 | 0.00 | **±** | 0.00 |  | **±** |  |  | **±** |  |  | **±** |  |  | **±** |  |  | **±** |  |  | **±** |  |  | **±** |  |
|  |  | 18 | 0.82 | **±** | 0.12 | 0.28 | **±** | 0.04 | 1.00 | **±** | 0.35 | 0.50 | **±** | 0.26 | 0.20 | **±** | 0.13 | 0.60 | **±** | 0.29 | 0.30 | **±** | 0.15 |  | **±** |  |  | **±** |  |  | **±** |  |  | **±** |  |  | **±** |  |  | **±** |  |  | **±** |  |
|  |  | 22 | 0.82 | **±** | 0.12 | 0.26 | **±** | 0.03 | 1.44 | **±** | 0.34 | 0.44 | **±** | 0.27 | 0.22 | **±** | 0.13 | 0.78 | **±** | 0.20 | 0.89 | **±** | 0.38 | -2.82 | **±** | 1.36 | 32.36 | **±** | 0.51 | 0.76 | **±** | 0.07 | 44.41 | **±** | 4.02 | 0.011 | **±** | 0.001 | 0.004 | **±** | 0.0002 | 0.004 | **±** | 0.001 |
|  | Extreme MHW | 8 | 1.00 | **±** | 0.00 | 0.36 | **±** | 0.04 | 0.90 | **±** | 0.28 | 0.10 | **±** | 0.10 | 0.70 | **±** | 0.21 | 0.50 | **±** | 0.27 | 0.50 | **±** | 0.27 |  | **±** |  |  | **±** |  |  | **±** |  |  | **±** |  |  | **±** |  |  | **±** |  |  | **±** |  |
|  |  | 12 | 0.90 | **±** | 0.10 | 0.45 | **±** | 0.03 | 1.70 | **±** | 0.45 | 0.00 | **±** | 0.00 | 0.40 | **±** | 0.16 | 0.60 | **±** | 0.27 | 0.50 | **±** | 0.27 |  | **±** |  |  | **±** |  |  | **±** |  |  | **±** |  |  | **±** |  |  | **±** |  |  | **±** |  |
|  |  | 18 | 0.00 | **±** | 0.00 | 0.09 | **±** | 0.03 | 4.00 | **±** | 0.00 | 0.17 | **±** | 0.13 | 0.00 | **±** | 0.00 | 0.50 | **±** | 0.26 | 0.50 | **±** | 0.26 | -9.15 | **±** | 0.92 |  | **±** |  |  | **±** |  |  | **±** |  |  | **±** |  |  | **±** |  |  | **±** |  |
| Palm Beach (central) | Ambient | 8 | 1.00 | **±** | 0.00 | 0.32 | **±** | 0.04 | 0.55 | **±** | 0.21 | 0.18 | **±** | 0.12 | 0.64 | **±** | 0.20 | 0.18 | **±** | 0.12 | 0.00 | **±** | 0.00 |  | **±** |  |  | **±** |  |  | **±** |  |  | **±** |  |  | **±** |  |  | **±** |  |  | **±** |  |
|  |  | 12 | 1.00 | **±** | 0.00 | 0.40 | **±** | 0.04 | 0.73 | **±** | 0.30 | 0.27 | **±** | 0.14 | 0.55 | **±** | 0.21 | 0.18 | **±** | 0.12 | 0.00 | **±** | 0.00 |  | **±** |  |  | **±** |  |  | **±** |  |  | **±** |  |  | **±** |  |  | **±** |  |  | **±** |  |
|  |  | 18 | 0.82 | **±** | 0.12 | 0.29 | **±** | 0.03 | 1.00 | **±** | 0.47 | 0.55 | **±** | 0.16 | 0.45 | **±** | 0.21 | 0.91 | **±** | 0.21 | 0.27 | **±** | 0.19 |  | **±** |  |  | **±** |  |  | **±** |  |  | **±** |  |  | **±** |  |  | **±** |  |  | **±** |  |
|  |  | 22 | 0.82 | **±** | 0.12 | 0.28 | **±** | 0.05 | 1.18 | **±** | 0.46 | 0.73 | **±** | 0.19 | 0.45 | **±** | 0.21 | 1.18 | **±** | 0.26 | 0.27 | **±** | 0.19 | -1.73 | **±** | 0.94 | 34.58 | **±** | 0.51 | 0.82 | **±** | 0.06 | 43.79 | **±** | 3.50 | 0.009 | **±** | 0.002 | 0.003 | **±** | 0.0002 | 0.003 | **±** | 0.001 |
|  | Strong MHW | 8 | 1.00 | **±** | 0.00 | 0.32 | **±** | 0.03 | 0.45 | **±** | 0.16 | 0.09 | **±** | 0.09 | 0.64 | **±** | 0.20 | 0.27 | **±** | 0.19 | 0.00 | **±** | 0.00 |  | **±** |  |  | **±** |  |  | **±** |  |  | **±** |  |  | **±** |  |  | **±** |  |  | **±** |  |
|  |  | 12 | 1.00 | **±** | 0.00 | 0.35 | **±** | 0.05 | 0.64 | **±** | 0.20 | 0.18 | **±** | 0.18 | 0.55 | **±** | 0.16 | 0.27 | **±** | 0.19 | 0.00 | **±** | 0.00 |  | **±** |  |  | **±** |  |  | **±** |  |  | **±** |  |  | **±** |  |  | **±** |  |  | **±** |  |
|  |  | 18 | 1.00 | **±** | 0.00 | 0.29 | **±** | 0.02 | 0.82 | **±** | 0.26 | 0.27 | **±** | 0.14 | 0.27 | **±** | 0.14 | 0.45 | **±** | 0.21 | 0.09 | **±** | 0.09 |  | **±** |  |  | **±** |  |  | **±** |  |  | **±** |  |  | **±** |  |  | **±** |  |  | **±** |  |
|  |  | 22 | 0.90 | **±** | 0.10 | 0.23 | **±** | 0.04 | 1.50 | **±** | 0.40 | 0.30 | **±** | 0.15 | 0.20 | **±** | 0.13 | 1.10 | **±** | 0.35 | 0.20 | **±** | 0.13 | -0.80 | **±** | 0.46 | 34.20 | **±** | 0.52 | 0.88 | **±** | 0.05 | 39.26 | **±** | 2.76 | 0.009 | **±** | 0.002 | 0.003 | **±** | 0.0001 | 0.003 | **±** | 0.001 |
|  | Extreme MHW | 8 | 1.00 | **±** | 0.00 | 0.39 | **±** | 0.06 | 0.33 | **±** | 0.17 | 0.00 | **±** | 0.00 | 0.56 | **±** | 0.18 | 0.11 | **±** | 0.11 | 0.00 | **±** | 0.00 |  | **±** |  |  | **±** |  |  | **±** |  |  | **±** |  |  | **±** |  |  | **±** |  |  | **±** |  |
|  |  | 12 | 0.89 | **±** | 0.11 | 0.39 | **±** | 0.06 | 1.00 | **±** | 0.50 | 0.00 | **±** | 0.00 | 0.44 | **±** | 0.18 | 0.22 | **±** | 0.15 | 0.00 | **±** | 0.00 |  | **±** |  |  | **±** |  |  | **±** |  |  | **±** |  |  | **±** |  |  | **±** |  |  | **±** |  |
|  |  | 18 | 0.11 | **±** | 0.11 | 0.17 | **±** | 0.04 | 3.43 | **±** | 0.50 | 0.29 | **±** | 0.16 | 0.14 | **±** | 0.13 | 0.86 | **±** | 0.23 | 0.29 | **±** | 0.25 | -2.75 | **±** | 0.86 |  | **±** |  |  | **±** |  |  | **±** |  |  | **±** |  |  | **±** |  |  | **±** |  |
| Cronulla (central) | Ambient | 8 | 1.00 | **±** | 0.00 | 0.33 | **±** | 0.04 | 0.42 | **±** | 0.15 | 0.08 | **±** | 0.08 | 0.67 | **±** | 0.22 | 0.00 | **±** | 0.00 | 0.00 | **±** | 0.00 |  | **±** |  |  | **±** |  |  | **±** |  |  | **±** |  |  | **±** |  |  | **±** |  |  | **±** |  |
|  |  | 12 | 0.92 | **±** | 0.08 | 0.42 | **±** | 0.06 | 1.00 | **±** | 0.37 | 0.17 | **±** | 0.11 | 0.50 | **±** | 0.15 | 0.00 | **±** | 0.00 | 0.00 | **±** | 0.00 |  | **±** |  |  | **±** |  |  | **±** |  |  | **±** |  |  | **±** |  |  | **±** |  |  | **±** |  |
|  |  | 18 | 0.83 | **±** | 0.11 | 0.36 | **±** | 0.04 | 0.80 | **±** | 0.27 | 0.20 | **±** | 0.12 | 0.20 | **±** | 0.12 | 0.30 | **±** | 0.14 | 0.10 | **±** | 0.09 |  | **±** |  |  | **±** |  |  | **±** |  |  | **±** |  |  | **±** |  |  | **±** |  |  | **±** |  |
|  |  | 22 | 0.75 | **±** | 0.13 | 0.29 | **±** | 0.03 | 1.20 | **±** | 0.40 | 0.30 | **±** | 0.14 | 0.20 | **±** | 0.12 | 0.60 | **±** | 0.24 | 0.40 | **±** | 0.28 | -0.68 | **±** | 0.51 | 33.28 | **±** | 0.99 | 0.95 | **±** | 0.06 | 35.74 | **±** | 2.31 | 0.009 | **±** | 0.001 | 0.003 | **±** | 0.0001 | 0.003 | **±** | 0.001 |
|  | Strong MHW | 8 | 1.00 | **±** | 0.00 | 0.37 | **±** | 0.03 | 0.25 | **±** | 0.13 | 0.17 | **±** | 0.11 | 0.50 | **±** | 0.15 | 0.08 | **±** | 0.08 | 0.00 | **±** | 0.00 |  | **±** |  |  | **±** |  |  | **±** |  |  | **±** |  |  | **±** |  |  | **±** |  |  | **±** |  |
|  |  | 12 | 1.00 | **±** | 0.00 | 0.38 | **±** | 0.04 | 0.25 | **±** | 0.13 | 0.25 | **±** | 0.13 | 0.17 | **±** | 0.11 | 0.08 | **±** | 0.08 | 0.00 | **±** | 0.00 |  | **±** |  |  | **±** |  |  | **±** |  |  | **±** |  |  | **±** |  |  | **±** |  |  | **±** |  |
|  |  | 18 | 1.00 | **±** | 0.00 | 0.38 | **±** | 0.03 | 0.58 | **±** | 0.26 | 0.67 | **±** | 0.14 | 0.08 | **±** | 0.08 | 0.25 | **±** | 0.13 | 0.08 | **±** | 0.08 |  | **±** |  |  | **±** |  |  | **±** |  |  | **±** |  |  | **±** |  |  | **±** |  |  | **±** |  |
|  |  | 22 | 1.00 | **±** | 0.00 | 0.31 | **±** | 0.02 | 0.75 | **±** | 0.28 | 0.92 | **±** | 0.15 | 0.08 | **±** | 0.08 | 0.75 | **±** | 0.28 | 0.08 | **±** | 0.08 | -0.33 | **±** | 0.38 | 33.11 | **±** | 0.73 | 0.96 | **±** | 0.05 | 34.78 | **±** | 1.64 | 0.011 | **±** | 0.002 | 0.004 | **±** | 0.0003 | 0.003 | **±** | 0.001 |
|  | Extreme MHW | 8 | 1.00 | **±** | 0.00 | 0.31 | **±** | 0.03 | 0.75 | **±** | 0.18 | 0.17 | **±** | 0.11 | 0.67 | **±** | 0.19 | 0.50 | **±** | 0.23 | 0.00 | **±** | 0.00 |  | **±** |  |  | **±** |  |  | **±** |  |  | **±** |  |  | **±** |  |  | **±** |  |  | **±** |  |
|  |  | 12 | 1.00 | **±** | 0.00 | 0.34 | **±** | 0.03 | 1.42 | **±** | 0.36 | 0.08 | **±** | 0.08 | 0.17 | **±** | 0.11 | 0.50 | **±** | 0.23 | 0.00 | **±** | 0.00 |  | **±** |  |  | **±** |  |  | **±** |  |  | **±** |  |  | **±** |  |  | **±** |  |  | **±** |  |
|  |  | 18 | 0.00 | **±** | 0.00 | 0.05 | **±** | 0.02 | 4.00 | **±** | 0.00 | 0.00 | **±** | 0.00 | 0.00 | **±** | 0.00 | 0.13 | **±** | 0.10 | 0.63 | **±** | 0.26 | -2.62 | **±** | 1.40 |  | **±** |  |  | **±** |  |  | **±** |  |  | **±** |  |  | **±** |  |  | **±** |  |

**Table S10: Differences in *Phyllospora comosa* juvenile survival (0-1) from different populations (Bonny Hills, Black Head, Palm Beach, Cronulla) and under three MHW scenarios (Ambient, strong and extreme MHW)** based on Generalised Linear Models (GLMs) with binomial distribution. Individual models were conducted after 12, 18 and 22 days. Differences were added for significant effects. Surv is used as an abbreviation for Survival.

| Response | Day | Factor | Differences | χ^2^ | df | p-value |
| --- | --- | --- | --- | --- | --- | --- |
| Survival | 12 | Population |  | 0.08 | 3 | 0.99 |
|  |  | MHW |  | 4.66 | 2 | 0.1 |
|  |  | Interaction |  | 4.78 | 6 | 0.57 |
|  |  |  |  |  |  |  |
|  | 18 | Population |  | 1.19 | 3 | 0.76 |
|  |  | MHW | Surv_Extreme_ < Surv_strong_ | 89.41 | 2 | **<0.001** |
|  |  |  | Surv_Extreme_ < Surv_ambient_ |  |  |  |
|  |  | Interaction |  | 12.15 | 6 | 0.06 |
|  | 22 | Population |  | 0.7 | 3 | 0.87 |
|  |  | MHW |  | 0.45 | 1 | 0.5 |
|  |  | Interaction |  | 7.8 | 3 | 0.051 |

**Table S11: Differences in *Phyllospora comosa* juvenile relative growth rate (RGR %d^-1^) from different populations (Bonny Hills, Black Head, Palm Beach, Cronulla) and under ambient and strong MHW scenarios** based on Linear Models (LMs). These data were only analysed for one time point (corresponding to the end of the experiment).

| Response | Factor |  | F | df | p-value |
| --- | --- | --- | --- | --- | --- |
| RGR | Population |  | 0.75 | 3 | 0.53 |
|  | MHW |  | 0.1 | 1 | 0.76 |
|  | Interaction | | 0.8 | 3 | 0.51 |

**Table S12: Differences in *Phyllospora comosa* juvenile Fv/Fm from different populations (Bonny Hills, Black Head, Palm Beach, Cronulla) and under three MHW scenarios (Ambient, strong and extreme MHW)** based on Linear Models (LMs). Individual models were conducted after 8, 12, 18 and 22 days. Differences were added for significant effects and only for significant interactions in case they occurred.

| Response | Day | Factor | Differences | F | df | p-value |
| --- | --- | --- | --- | --- | --- | --- |
| Fv/Fm | 8 | Population |  | 0.01 | 3 | 1 |
|  |  | MHW |  | 0.01 | 2 | 1 |
|  |  | Interaction |  | 1.14 | 6 | 0.35 |
|  | 12 | Population |  | 0.24 | 3 | 0.87 |
|  |  | MHW |  | 0.45 | 2 | 0.64 |
|  |  | Interaction |  | 0.44 | 6 | 0.85 |
|  | 18 | Population |  | 1.44 | 3 | 0.24 |
|  |  | MHW | Fv/Fm_extreme_ < Fv/Fm_strong_ | 44.7 | 2 | **< 0.001** |
|  |  |  | Fv/Fm_extreme_ < Fv/Fm_ambient_ |  |  |  |
|  |  | Interaction |  | 2.13 | 6 | 0.06 |
|  | 22 | Population |  | 0.49 | 3 | 0.7 |
|  |  | MHW |  | 2.09 | 1 | 0.15 |
|  |  | Interaction |  | 1.36 | 3 | 0.26 |

**Table S13: Differences in *Phyllospora comosa* juvenile structural integrity loss (0-4) from different populations (Bonny Hills, Black Head, Palm Beach, Cronulla) and under three MHW scenarios (Ambient, strong and extreme MHW)** based on Linear Models (LMs). Individual models were conducted after 8, 12, 18 and 22 days. Differences were added for significant effects. SIL is used as an abbreviation for structural integrity loss

| Response | Day | Factor | Differences | F | df | p-value |
| --- | --- | --- | --- | --- | --- | --- |
| Structural integrity loss | 8 | Population |  | 1.31 | 3 | 0.27 |
|  |  | MHW |  | 1.93 | 2 | 0.15 |
|  |  | Interaction |  | 1.28 | 6 | 0.27 |
|  | 12 | Population |  | 8.8 | 3 | 0.45 |
|  |  | MHW | SIL_extreme_ > SIL_strong_ | 3.9 | 2 | **0.02** |
|  |  | Interaction |  | 1.15 | 6 | 0.34 |
|  | 18 | Population |  | 0.79 | 3 | 0.54 |
|  |  | MHW | SIL_extreme_ > SIL_strong_ | 67.86 | 2 | **<0.001** |
|  |  |  | SIL_extreme_ > SIL_ambient_ |  |  |  |
|  |  | Interaction |  | 0.47 | 6 | 0.83 |
|  | 22 | Population |  | 0.56 | 3 | 0.64 |
|  |  | MHW |  | 0.26 | 1 | 0.61 |
|  |  | Interaction |  | 0.93 | 3 | 0.43 |

**Table S14: Differences in *Phyllospora comosa* juvenile fouling level (0-4) from different populations (Bonny Hills, Black Head, Palm Beach, Cronulla) and under three MHW scenarios (Ambient, strong and extreme MHW)** based on Linear Models (LMs). Individual models were conducted after 8, 12, 18 and 22 days. Differences were added for significant effects.

| Response | Day | Factor | Differences | F | df | p-value |
| --- | --- | --- | --- | --- | --- | --- |
| Fouling | 8 | Population |  | 0.86 | 3 | 0.47 |
|  |  | MHW |  | 0.99 | 2 | 0.38 |
|  |  | Interaction |  | 0.35 | 6 | 0.91 |
|  | 12 | Population |  | 2.32 | 3 | 0.08 |
|  |  | MHW |  | 3.05 | 2 | 0.051 |
|  |  | Interaction |  | 0.69 | 6 | 0.66 |
|  | 18 | Population |  | 1.81 | 3 | 0.15 |
|  |  | MHW | Fouling_extreme_ < Fouling_ambient_ | 4.96 | 2 | **<0.01** |
|  |  | Interaction |  | 0.54 | 6 | 0.77 |
|  | 22 | Population |  | 0.64 | 3 | 0.59 |
|  |  | MHW |  | 2.41 | 1 | 0.13 |
|  |  | Interaction |  | 1.05 | 3 | 0.38 |

**Table S15: Differences in *Phyllospora comosa* juvenile bleaching level (0-4) from different populations (Bonny Hills, Black Head, Palm Beach, Cronulla) and under three MHW scenarios (Ambient, strong and extreme MHW)** based on Linear Models (LMs). Individual models were conducted after 8, 12, 18 and 22 days. Differences were added for significant effects and only for significant interactions in case they occurred. Bl is used as an abbreviation for bleaching.

| Response | Day | Factor | Differences | F | df | p-value |
| --- | --- | --- | --- | --- | --- | --- |
| Bleaching | 8 | Population |  | 2.2 | 3 | 0.09 |
|  |  | MHW |  | 0.16 | 2 | 0.85 |
|  |  | Interaction |  | 2.09 | 6 | 0.06 |
|  | 12 | Population |  | 1.13 | 3 | 0.34 |
|  |  | MHW |  | 0.95 | 2 | 0.39 |
|  |  | Interaction |  | 0.53 | 6 | 0.78 |
|  | 18 | Population |  | 0.45 | 3 | 0.72 |
|  |  | MHW |  | 2.47 | 2 | 0.09 |
|  |  | Interaction |  | 1.13 | 6 | 0.35 |
|  | 22 | Population |  | 0.36 | 3 | 0.78 |
|  |  | MHW |  | 0.22 | 1 | 0.64 |
|  |  | Interaction | Bl_strong-Cronulla_ > Bl_ambient-Cronulla_ | 2.87 | 3 | **0.04** |

**Table S16: Differences in *Phyllospora comosa* juvenile necrosis (0-4) from different populations (Bonny Hills, Black Head, Palm Beach, Cronulla) and under three MHW scenarios (Ambient, strong and extreme MHW)** based on Linear Models (LMs). Individual models were conducted after 8, 12, 18 and 22 days. Differences were added for significant effects.

| Response | Day | Factor | Differences | F | df | p-value |
| --- | --- | --- | --- | --- | --- | --- |
| Necrosis | 8 | Population |  | 0.89 | 3 | 0.45 |
|  |  | MHW |  | 2.56 | 2 | 0.08 |
|  |  | Interaction |  | 1.41 | 6 | 0.21 |
|  | 12 | Population |  | 0.39 | 3 | 0.76 |
|  |  | MHW | Necrosis_extreme_ > Necrosis_ambient_ | 3.27 | 2 | **0.04** |
|  |  | Interaction |  | 1.73 | 6 | 0.12 |
|  | 18 | Population | Necrosis_Palm Beach_ > Necrosis_Cronulla_ | 2.92 | 3 | **0.04** |
|  |  | MHW |  | 0.21 | 2 | 0.81 |
|  |  | Interaction |  | 1.02 | 6 | 0.42 |
|  | 22 | Population |  | 1.22 | 3 | 0.31 |
|  |  | MHW |  | 1.34 | 1 | 0.25 |
|  |  | Interaction |  | 0.79 | 3 | 0.50 |

**Table S17: Differences in *Phyllospora comosa* juvenile green colouration (0-4) from different populations (Bonny Hills, Black Head, Palm Beach, Cronulla) and under three MHW scenarios (Ambient, strong and extreme MHW)** based on Linear Models (LMs). Individual models were conducted after 8, 12, 18 and 22 days. Differences were added for significant effects and only for significant interactions in case they occurred. GC is used as an abbreviation for green colouration

| Response | Day | Factor | Differences | F | df | p-value |
| --- | --- | --- | --- | --- | --- | --- |
| Green colouration | 8 | Population |  | 3.51 | 3 | **0.02** |
|  |  | MHW |  | 3.41 | 2 | **0.04** |
|  |  | Interaction | GC_extreme-Black Head_ > GC_ambient-Black Head_ | 3.44 | 6 | **<0.01** |
|  |  |  | GC_extreme-Black Head_ > GC_strong-Black Head_ |  |  |  |
|  |  |  | GC_extreme-Black Head_ > GC_extreme-Bonny Hills_ |  |  |  |
|  |  |  | GC_extreme-Black Head_ > GC_extreme-Palm Beach_ |  |  |  |
|  |  |  | GC_extreme-Black Head_ > GC_extreme-Cronulla_ |  |  |  |
|  | 12 | Population |  | 3.46 | 3 | 0.02 |
|  |  | MHW |  | 3.56 | 2 | 0.03 |
|  |  | Interaction | GC_extreme-Black Head_ > GC_ambient-Black Head_ | 3.52 | 6 | **<0.01** |
|  |  |  | GC_extreme-Black Head_ > GC_strong-Black Head_ |  |  |  |
|  |  |  | GC_extreme-Black Head_ > GC_extreme-Bonny Hills_ |  |  |  |
|  |  |  | GC_extreme-Black Head_ > GC_extreme-Palm Beach_ |  |  |  |
|  |  |  | GC_extreme-Black Head_ > GC_extreme-Cronulla_ |  |  |  |
|  | 18 | Population |  | 0.9 | 3 | 0.45 |
|  |  | MHW | GC_extreme_ > GC_ambient_ | 7.7 | 2 | **<0.001** |
|  |  |  | GC_extreme_ > GC_strong_ |  |  |  |
|  |  | Interaction |  | 1.7 | 6 | 0.13 |
|  | 22 | Population |  | 1.56 | 3 | 0.21 |
|  |  | MHW |  | 0.02 | 1 | 0.9 |
|  |  | Interaction |  | 0.71 | 3 | 0.55 |

**Table S18: Differences in *Phyllospora comosa* juvenile C:N ratio, and tissue carbon (C) and nitrogen (N, %) from different populations (Bonny Hills, Black Head, Palm Beach, Cronulla) and under ambient and strong MHW scenarios** based on Linear Models (LMs). These data were only analysed for one time point (corresponding to the end of the experiment). Differences were added for significant effects and only for significant interactions in case they occurred.

| Response | Factor | Differences | F | df | p-value |
| --- | --- | --- | --- | --- | --- |
| C:N ratio | Population |  | 1.84 | 3 | 0.16 |
|  | MHW |  | 1.59 | 1 | 0.22 |
|  | Interaction |  | 2.17 | 3 | 0.11 |
| Tissue C | Population | C_Palm Beach_ > C_Black Head_ | 4.77 | 3 | **<0.01** |
|  |  | C_Palm Beach_ > C_Bonny Hills_ |  |  |  |
|  | MHW |  | 0.26 | 1 | 0.62 |
|  | Interaction |  | 0.35 | 3 | 0.79 |
| Tissue N | Population |  | 2.65 | 3 | 0.06 |
|  | MHW |  | 1.31 | 1 | 0.25 |
|  | Interaction |  | 1.84 | 3 | 0.14 |

**Table S19: Differences in *Phyllospora comosa* juvenile pigment content (chlorophyll-a, chlorophyll-c and fucoxanthin, mg g^-1^) from different populations (Bonny Hills, Black Head, Palm Beach, Cronulla) and under ambient and strong MHW scenarios** based on Linear Models (LMs). These data were only analysed for one time point (corresponding to the end of the experiment). Differences were added for significant effects and only for significant interactions in case they occurred

| Response | Factor | Differences | F | df | p-value |
| --- | --- | --- | --- | --- | --- |
| Chlorophyll-a | Population |  | 0.2 | 3 | 0.89 |
|  | MHW |  | 1.62 | 1 | 0.21 |
|  | Interaction |  | 0.32 | 3 | 0.81 |
| Chlorophyll-c | Population |  | 0.85 | 3 | 0.47 |
|  | MHW | Chl-c_Ambient_ < Chl-c_strong_ | 9.32 | 1 | **<0.01** |
|  | Interaction |  | 0.53 | 3 | 0.66 |
| Fucoxanthin | Population |  | 0.45 | 3 | 0.72 |
|  | MHW |  | 0.84 | 1 | 0.36 |
|  | Interaction |  | 0.31 | 3 | 0.82 |
